# Chitosan-PEG Gels Loaded with *Jatropha mollissima* (Pohl) Baill. Ethanolic Extract: An Efficient and Effective Biomaterial in Hemorrhage Control

**DOI:** 10.3390/ph16101399

**Published:** 2023-10-03

**Authors:** José F. B. Rodrigues, João V. S. de A. Queiroz, Rebeca P. Medeiros, Rafaela O. Santos, Djair A. Fialho, João E. S. Neto, Rogério L. dos Santos, Rossemberg C. Barbosa, Wladymyr J. B. Sousa, Maria da C. de M. Torres, Luanna A. D. M. Medeiros, Suédina M. de L. Silva, Maziar Montazerian, Marcus V. L. Fook, Solomon K. S. Amoah

**Affiliations:** 1 Materials Science and Engineering Department, Northeast Laboratory for Evaluation and Development of Biomaterials, Academic Unit of Materials Science and Engineering, Federal University of Campina Grande, Campina Grande 58429-000, PB, Brazilmariatorres@servidor.uepb.edu.br (M.d.C.d.M.T.); suedina.lima@certbio.ufcg.edu.br (S.M.d.L.S.); maziar_montaz@yahoo.com (M.M.); marcus.liafook@certbio.ufcg.edu.br (M.V.L.F.); 2 Department of Dentistry, Life Science Institute, Federal University of Juiz de Fora, Governador Valadares 36036-900, MG, Brazil; 3 Chemistry Department, Science and Technology Center, State University of Paraiba, Campina Grande 58429-500, PB, Brazil; 4 Brazilian Association of Support Cannabis Esperança, João Pessoa 58013-130, PB, Brazil

**Keywords:** chitosan, plant extracts, injuries, bleeding, biomaterials, hemostasis

## Abstract

A lack of control over blood loss can have catastrophic implications, including death. Although several hemostatic medications have been employed to reduce bleeding, a vast majority of them are ineffective, expensive, or pose health risks to the patient. To overcome these constraints, chitosan-polyethylene glycol (CS-PEG) hemostatic gels loaded with ethanolic extract of *Jatropha mollissima* sap (EES) were prepared and their hemostatic, physicochemical, and cytotoxic properties were evaluated. The gels were produced by mixing CS with PEG (an external plasticizer) and EES. The phytochemical analysis revealed a significant concentration of total polyphenols and tannins content in the extract and catechin was identified as one of the key compounds of EES. Infrared spectroscopy analysis revealed the presence of EES in the gels, as well as the chemical interaction between CS and PEG. The gels were thermally stable between 25 and 37 °C (ambient and human body temperature range), had pseudoplastic deformation behavior (rheological properties preserved after shearing), were simple to inject (compression force 30 N), and were biocompatible. In vivo experiments showed that both CS-PEG-EES gels exhibited greater hemostatic action in preventing tail hemorrhage in Wistar rats, with decreased bleeding time and blood weight compared with unloaded CS-PEG gels (control groups) and Hemostank, a commercial product. However, the gel prepared with acetic acid was more efficient in controlling bleeding. These findings reveal that CS-PEG-EES gels can reduce hemorrhages and are a potent, simple, and safe hemostatic agent.

## 1. Introduction

Hemorrhage-related blood loss is the second greatest cause of death in hospitals [1]. Traumatic injury is responsible for about 5.8 million deaths annually [2]. Suture, gauze compression, the elevation of the bleeding point, and the tourniquet have all been employed to control bleeding over the years. These approaches are helpful to some extent, but they do not perform well when the patient has comorbidities, treating deep and irregular lesions, and/or when other risks, such as infections, are present [3]. As a result, biomaterials capable of providing hemostasis have been proposed as an alternative to traditional procedures.

The most common hemostatic materials on the market include collagen, gelatin, alginate, chitosan, porous zeolite, and carboxymethylcellulose [4]. While all of them are effective in controlling hemostasis, they are often expensive or can cause health concerns in the patient [5]. Collagen has limited hemostatic activity as it only relies on platelet activation, and despite being biocompatible and biodegradable, it can trigger allergic/immune reactions [6]. Porous zeolite, on the other hand, may cause wound inflammation and tissue necrosis [7], while carboxymethylcellulose dressings do not degrade and cause scarring when removed [4]. As a result, although breakthroughs in hemostatic drug development have been made, there is still a need for new low-cost and safe treatments that can effectively control bleeding.

Chitosan (CS) is a renewable, low-cost, and biocompatible biopolymer with hemostatic properties commonly used to produce hemostatic products [8,9]. Numerous CS-based hemostatic medicines have been approved by the Food and Drug Administration (FDA) and are commercially accessible. Celox^®^ (gauze, swab, and powder), Chitodine^®^ (powder), TraumaStat^®^ (spray), Chitoseal^®^ and XStat^®^ (gels), HemCon^®^ and Chitoflex^®^ Bandages (patches and swabs), QuikClot^®^ (gauze, compresses, and sponges), and Clo-Sur^®^ (compresses) are only a few examples [4]. However, the intrinsic stiffness and fragility of pure CS result in inadequate mechanical strength of these biomaterials, which limits CS as a hemostatic agent [10].

CS has been combined with various polymers and hemostatic agents to compensate for these limitations [8]. Polyethylene glycol (PEG) is a biodegradable hydrophilic synthetic polymer with hemostatic activity, which is used to improve the biocompatibility [11] and mechanical properties of a variety of biomaterials, such as scaffolds, films, gels, and so on [12]. Hydrogels based on CS and PEG have better physiochemical properties. Because of the hydroxyl groups at their ends, PEG provides higher mobility to gels when coupled with CS [8]. Some researchers have reported improvements in CS’s mechanical [13] and biological properties after adding PEG [14,15].

Plant extracts have been widely employed as hemostatic agents, either topically or in the production of biomaterials [16]. *Jatropha mollissima* (Pohl) Baill. is a plant in the genus *Jatropha* commonly referred to as “Pinhão-bravo.” It is easily cultivated and has antiophidic, anti-inflammatory, and hemostatic characteristics, acting on protein precipitation [17]. In our previous study, we found that an ethanolic extract of *Jatropha mollissima* sap (EES) had hemostatic action in Wistar rats [18]. EES showed hemostatic activity at both concentrations (25 and 40 mg·mL^−1^), with the 25 mg·mL^−1^ concentration yielding the best results as it did not cause rebleeding and resulted in a hemostasis time 1.72 times faster and blood volume 6 times less than Monsel^®^. Based on these findings, EES may enhance the hemostatic activity of CS-based biomaterials by a synergistic action during the coagulation cascade, with CS acting on platelet aggregation and EES working on protein precipitation.

In this study, hemostatic healing gels of CS-PEG-EES were produced and their physical-chemical, hemostatic, and cytotoxic properties were examined to determine whether the CS loaded with EES improves its hemostatic activity. Hereafter, we show that CS gels, prepared by combining CS with various amounts of PEG in the presence of EES, are not cytotoxic to L929 cells, possess appropriate mechanical characteristics, and effectively reduce bleeding times and blood weight compared with the control groups and Hemostank. The findings demonstrate that CS-PEG-EES gels are effective and capable of reducing bleeding and can be utilized to control internal, external, and local hemorrhages.

## 2. Results and Discussion

### 2.1. Phytochemical Analysis

The phytochemical study identified a key component of the ethyl acetate fraction (EAF). When analyzed by thin layer chromatography (TLC) with UV (254 nm) and phosphoric vanillin and heated, the fraction corresponding to the mixture of containers 55 to 60 (fraction A9) revealed bright purple and reddish-orange colors, respectively (Figure S1, Supplementary Materials). Its mass spectrum showed multiple molecular ions [M–H]^−^ at 289.08, 311.17, 325.19, and 339.21 *m*/*z*, with the most abundant being 289.08 *m*/*z* (Figure S2, Supplementary Materials). This confirmed that the isolated substance was most likely not pure.

The hydrogen Nuclear Magnetic Resonance (NMR) spectrum (Figure S3, Supplementary Materials) showed doublets at 4.47 ppm (1H, J = 7.2 Hz); 5.87 ppm (1H, J = 2.3 Hz); 5.68 ppm (1H, J = 2.3 Hz); 6.70 ppm (1H, J = 2.3 Hz); 6.67 ppm (1H, J = 8.0 Hz); and 4.90 ppm (1H, J = 5.2 Hz) that were attributed to H-2, H-6, H-8, H-2′, H-5′, and 3-OH, respectively. Double of doublets were found at 2.64 ppm (1H, J = 16.0; 5.2 Hz); 2.34 ppm (1H, J = 16.0; 8.0 Hz); and 6.58 ppm (1H, J = 8.0; 2.3 Hz), which were attributed to H-4a, H-4b, and H-6′, respectively. A multiplet at 3.80 ppm (1H) was attributed to H-3; and singlets at 9.15 ppm (1H), 8.91 ppm (1H), 8.84 ppm (1H), and 8.79 ppm (1H) were detected and attributed to the hydroxyl hydrogens 5-OH, 7-OH, 4′-OH, and 3′-OH, respectively.

In the carbon spectrum (Figure S4, Supplementary Materials), signals with chemical shifts of 81.42 ppm, 66.74 ppm, 28.26 ppm, 156.62 ppm, 95.58 ppm, 156.88 ppm, 94.31 ppm, 155.79 ppm, 99.54 ppm, 131.02 ppm, 114.94 ppm, 144.88 ppm, 145.29 ppm, 115.53 ppm, and 118.90 ppm were assigned to carbons C-2, C-3, C-4, C-5, C-6, C-7, C-8, C-9, C-10, C-1′, C-2′, C-3′, C-4′, C-5′ and C-6′, respectively.

The FTIR spectrum (Figure S5, Supplementary Materials) revealed vibration bands in 3212 cm^−1^ (–OH); 2957, 2933, and 2854 cm^−1^ (saturated and unsaturated –CH stretching vibrations); 1624 cm^−1^ (aromatic C=C stretching bonds); 1281 cm^−1^ (aliphatic C–O bonds); and 1084 cm^−1^ (aromatic C–O bonds), both from the C–OH radical; and 670–450 cm^−1^ (C–H bending vibrations from the methylene radical). The extracted compound present in fraction A9 was identified as catechin based on MS [19], NMR [20,21,22,23], and FTIR data [24,25]. For better comparison, Table 1 compares ^13^C and ^1^H NMR data to the literature data.

**Figure 1:**
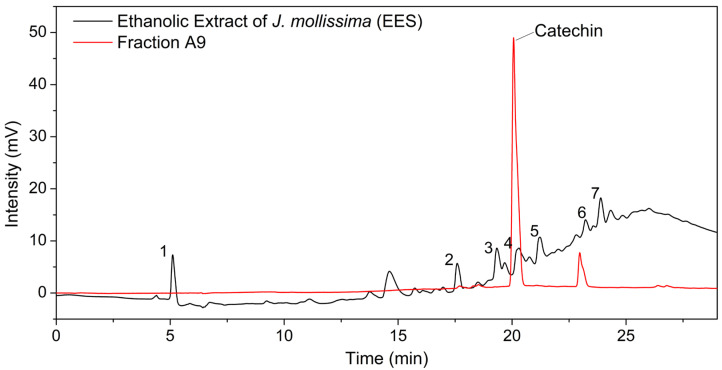
HPLC–DAD chromatogram of EES extract from *Jatropha mollissima* and fraction A9. Performed at 280 nm.

The methodology devised in HPLC–DAD to examine the EES extract allowed the separation of the extract’s main constituents. Fraction A9 was evaluated using this methodology, which separated two major compounds, one with a retention time of 20.06 min and the other with a retention time of 23 min (Figure 1), indicating a purity of approximately 80% for catechin (Table S1, Supplementary Materials). By comparing the retention time of the EES chromatograms and fraction A9, it was observed that catechin has the same retention time as the major compound number 4 identified in the EES. These results suggested that catechin is one of the key compounds present in the EES.

This is the first time that catechin has been isolated from *Jatropha mollissima*. Other compounds previously identified by some authors include spinasterol, triacontane [26], and the flavonoids isoschaftoside, schaftoside, isoorientin, orientin, vitexin, and isovitexin [27]. Researchers have identified various groups of secondary metabolites present in *Jatropha mollissima*, including coumarins, phenols, tannins, flavanols, flavanones, alkaloids, steroids, and triterpenoids [28].

Catechin is a flavonoid that possesses antioxidant and anti-inflammatory properties, promotes skin regeneration, and has been found in *Jatropha gossypiifolia* [29,30].

### 2.2. Total Concentration of Phenols and Condensed Tannins

The total concentrations of phenolics and condensed tannins present in the EES are shown in Table 2.

According to Table 2, the presence of polyphenols and tannins was confirmed in the EES. Tannins showed the highest concentration (512.30 mg·g^−1^) followed by polyphenols (242.84 mg·g^−1^). Polyphenols are compounds known for their anti-inflammatory, antioxidant, hemostatic, and procoagulant activities [31,32]. Tannins, in turn, stand out for their hypotensive activity, vasoconstrictive and astringent actions, and consequent hemostatic effect [33]. Mouffouk, Mouffouk, Oulmi, Mouffouk and Haba [34] evaluated the photoprotective, anti-inflammatory, antioxidant, and hemostatic properties of the methanolic extract of *Linaria scariosa*. The hemostatic and procoagulant activity of the extract was attributed mainly to the concentration of polyphenols in the composition (88.66 μg·mg^−1^). Additionally, studies point out that plants of the genera *Acacia*, *Asplenium*, *Capsela*, *Rubus* and *Terminalia* are rich in tannins and, for this reason, are used for hemostatic purposes [35]. One example is the species *Capsella bursa-pastoris*, which has anti-hemorrhagic properties associated with the presence of tannins [36]. Therefore, the hemostatic effect presented by EES in our last study [18] may be associated with the high concentration of polyphenols and tannins present in it.

### 2.3. Chemical Characterization of Raw Materials and CS-PEG-EES Gels

**Figure 2:**
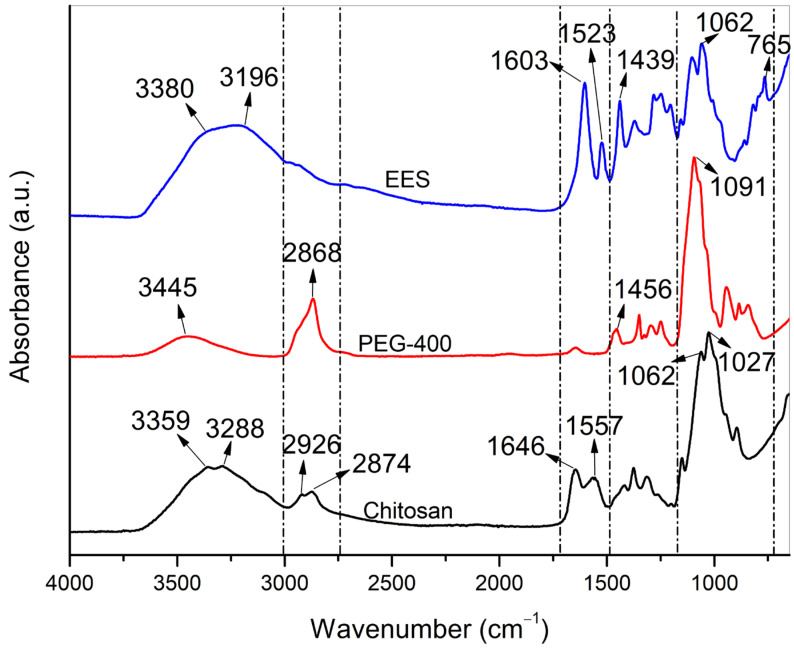
FTIR spectra of the raw materials used in making the gels—EES, PEG and CS.

The spectra of the raw materials used in making the gels are illustrated in Figure 2.

Figure 2 shows the distinctive bands of the CS spectrum, which correspond to the stretching vibrations of −OH and −NH_2_. Peaks at 2926 and 2874 cm^−1^ refer to the C−H stretches of the methylene group, while vibration at 1646 cm^−1^ belongs to the acetyl group of the primary amide, and the peak at 1557 cm^−1^ is associated with the −NH_3_ bond of the secondary amide [37]. The bands at 1061 and 1027 cm^−1^ are attributed to the C−O−C bonds typical of the glycosidic link between carbon 1 and 4 of the saccharide structure of CS [38]. PEG has a distinctive band at 1091 cm^−1^ from the ether group (−C−O−C−) [39]. Additionally, we can also observe the band at 3445 cm^−1^ that is associated with the hydroxyl group, the −CH group in stretching mode with the vibration at 2868 cm^−1^, and its folding modulus between 1460 and 1290 cm^−1^ [1].

The EES spectrum exhibits vibrational bands at 3380, 3196, and 1439 cm^−1^, corresponding to the −OH groups of phenolic compounds, as well as deformations between 3400 and 3200 cm^−1^ and peaks between 1450 and 1350 cm^−1^, referring to hydroxyl (−OH) stretching, which suggests the presence of alcohols and phenols [40]. The vibrations between 800 and 765 cm^−1^ correspond to the −CH bends of aromatic rings, most likely of terpene chains [41].

Additionally, we can observe bands between 1600 and 1500 cm^−1^, which are characteristic of the C=C group of flavonoids [42]. This group includes compounds like quercitrin, which has a hemostatic effect and has already been identified in *Jatropha gossypifolia* [29]. Some of the organic groups described, such as flavonoids and phenols, have already been identified as part of the chemical composition of *J. mollissima* [17], along with other groups such as tannins, which were found in high concentrations in EES.

**Figure 3:**
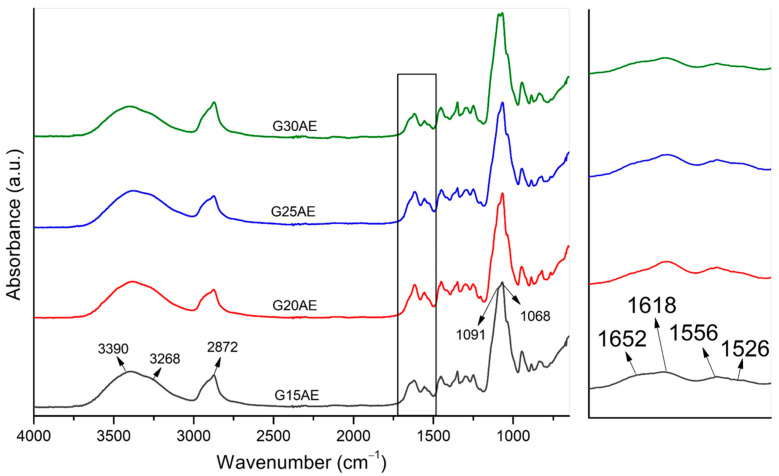
FTIR spectra of CS-PEG-EES gels prepared with acetic acid—G15AE; G20AE; G25AE; and G30AE (**left**) and spectra zoom in the range of 1670−1480 cm^−1^ (**right**).

Figure 3 shows the spectrum of gels produced in acetic acid with PEG fractions of 15, 20, 25, and 30% (*v*/*v*).

Figure 3 depicts that the main vibration bands of CS, such as the hydroxyl folds, amides, and saccharide groups, were preserved. The overlapping of the CS and EES peaks causes distortion in the bandwidth between 3600 and 3200 cm^−1^. Figure 3 zoom shows that, in addition to the peaks related to the acetyl group and the −NH_2_ group of CS, the EES peaks at 1618 and 1526 cm^−1^ are also present. The vibration at 2872 cm^−1^, which refers to the −CH group of PEG, appears rounded due to the presence of the methylene group. The peak at 1091 cm^−1^, which is also characteristic of PEG, formed a double peak due to the ether group of CS and characteristic bands of the EES that vibrate in the same regions [37,43]. The acetyl group was also shifted to higher wavelengths (from 1646 to 1652 cm^−1^) due to the formation of hydrogen bonds between the amide of the CS and the ether groups of PEG. These findings support the interaction of PEG with CS, which acts as an external plasticizer, creating weak second-order interactions, similar to the hydrogen bonds generated between the amide of CS and ether groups of PEG [44,45].

**Figure 4:**
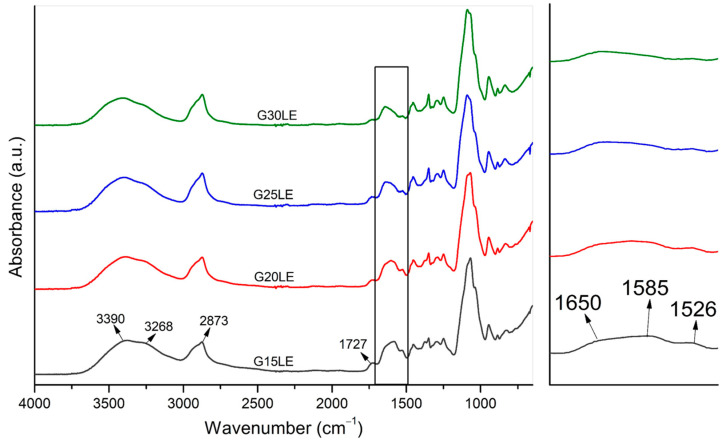
FTIR spectra of CS-PEG-EES gels prepared with lactic acid—G15LE; G20LE; G25LE; and G30LE (**left**) and spectra zoom in the range of 1670−1480 cm^−1^ (**right**).

The spectra of gels prepared with PEG fractions of 15, 20, 25, and 30% (*v*/*v*) and lactic acid are shown in Figure 4. The figure confirms the presence of bands corresponding to the hydroxyl, ethylene, and ether groups of CS in all of the samples, with the main differences being observed in the amide groups (Figure 4 zoom). The secondary amide peak was shifted to a higher wavelength (from 1557 cm^−1^ to 1585 cm^−1^) due to the lactic acid’s higher dissociation constant compared with that of acetic acid, which promotes more intense interaction between amines and the carboxylic groups and, as a result, decreases the intensity of the secondary amide band (−NH_2_). There was also a slight shift in the wavenumber of the acetyl group, which was pushed to a higher wavenumber (from 1646 to 1650 cm^−1^) due to the formation of hydrogen bonds between the amide of CS and the ether groups of PEG, as observed in the samples prepared with acetic acid. These shifts caused an overlap of the primary amide bands, the 1603 cm^−1^ peak of the EES, and the secondary amide, generating a rounded band. In Figure 4 zoom, it is still possible to observe that the 1526 cm^−1^ band referring to the EES remained unchanged. Furthermore, a signal at 1727 cm^−1^ was formed, indicating the presence of free carboxyls [43].

### 2.4. Rheological Analysis of CS-PEG-EES Gels

**Figure 5:**
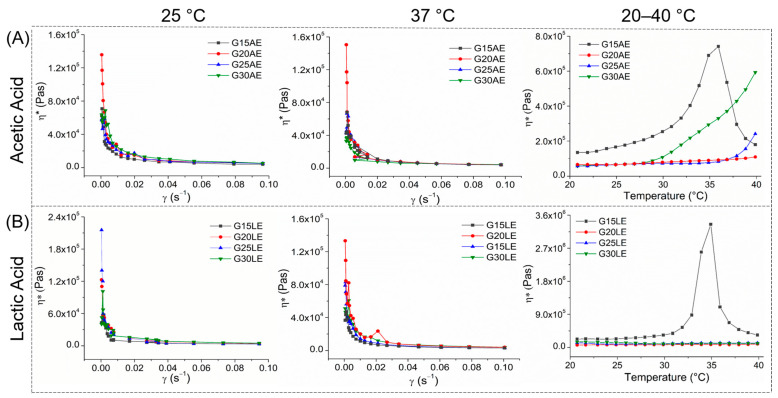
Viscosity curves (η*) vs. shear stress (γ) (0.001–0.1 (s^−1^)) at different temperatures (°C) (25, 37, and 20–40 °C) of the gels synthesized using (**A**) acetic acid, and (**B**) lactic acid.

The rheological analysis was performed to assess the apparent viscosity and viscoelastic behavior of the gels when subjected to increasing shear rates at fixed temperatures (25 and 37 °C) to determine the mechanical and flow properties at ambient and body temperature, respectively [46]. Figure 5 illustrates the relationship between the viscosity (η*) and shear rate (γ) of the gels prepared with acetic and lactic acid at 25 °C and 37 °C.

Figure 5 shows that all samples exhibited similar behavior when subjected to shear at temperatures ranging from 25 to 37 °C. They demonstrated a decrease in apparent viscosity as the shear rate increased, which is typical of non-Newtonian fluids with pseudoplastic deformation behavior. This property is appropriate for the use of hydrogels in human body applications because they become more fluid in situations of friction (during application), facilitating their dissemination [47].

The elastic (G′) and plastic (G″) loss modules of the gels (Figures S8–S11, Supplementary Materials) behaved similarly to the apparent viscosity, dropping proportionally with the increase in shear rate. Furthermore, the G′ remained greater than the G″, indicating that the gels tend to behave like a solid and do not change their physical state during shearing. As observed by Fang, Yang, Hong and Hu [48], the absence of changes in the thermal stability and solid-state of the gel is a significant behavior, since it suggests good stability of the gel’s structure, which contributes to hemostatic applications. This is an intriguing conclusion, considering that the material must buffer the bleeding to prevent blood flow [46].

### 2.5. Thermal Stability of CS-PEG-EES Gels

Thermal stability tests were conducted to evaluate the stability of the gels over a wide temperature range. Figure 5 depicts the correlation between viscosity (η*) and temperature for the compositions prepared with acetic and lactic acids.

The results of the acetic acid samples (Figure 5A) revealed consistent viscosity values between 20 and 28 °C (samples G20AE, G25AE, and G30AE) and a gradual increase with temperature (29 to 40 °C). The G15AE sample was an exception, exhibiting a viscosity peak at 37 °C. This viscosity characteristic suggests that the samples are heat sensitive, becoming more viscous as temperatures rise. The order of viscosity for the gels was G15AE > G30AE > G25AE > G20AE.

The lactic acid samples (Figure 5B) demonstrated consistent viscosity as the temperature increased. The viscosity of G20LE, G25LE, and G30LE samples remained virtually unchanged over the heating ramp, indicating that their flow properties are stable and unaffected by temperature fluctuations [49]. The G15LE sample deviated from this pattern, exhibiting the largest viscosity peak among all samples near 37 °C. The order of viscosity for the gels was G15LE > G30LE > G25LE > G20LE.

The high amount of PEG is responsible for the variation in viscosity between samples with 20, 25, and 30% PEG produced with both types of acid. The quantity of PEG promotes the formation of cross-links (such as hydrogen bonds) between its molecules, increasing gel viscosity [50]. This effect was more apparent in the compositions prepared with acetic acid.

The influence of acid type on gel viscosity is attributed to the size of their chains [51]. Acetic acid has one less methyl group (−CH_3_) than lactic acid. This allows the PEG to align itself over the CS chains, leading to an increase in gel viscosity [52]. Lactic acid, on the other hand, has a larger molecular structure, which increases the distance between the CS chains. This enables the PEG to situate itself between the CS chains, resulting in greater mobility and less cross-linking between the PEG chains [53,54].

Despite increasing the spacing between the CS and PEG chains, the apparent viscosity of the lactic acid compositions was greater than that of the acetic acid compositions. This is due to the fact that lactate ions are more voluminous than acetate ions, which increases the stiffness of the CS chains by making their organization difficult when the gel is deformed [51,55]. As a result, increasing the stiffness of the CS chains leads to an increase in viscosity. These findings are consistent with those of earlier research that studied the effects of carboxylic acids [51], and organic and inorganic acids [55,56,57] on the mechanical properties of chitosan fibers, biofilms, and gels.

### 2.6. Injectable Properties of CS-PEG-EES Gels

The FDA requires that human medicine and biological compositions be evaluated for injectability. This requirement is more stringent when these materials are in the form of gel and/or can be administered through an injection system, such as syringes. The injectability assay assesses the force needed to initiate and maintain movement of the plunger along the barrel of the syringe while the material flows at a constant rate [58]. When in gel form, the biomaterial can be topically administered to skin injuries as well as external and internal injuries. Therefore, due to these potential applications, injectability tests were conducted on CS-PEG-EES gels.

**Figure 6:**
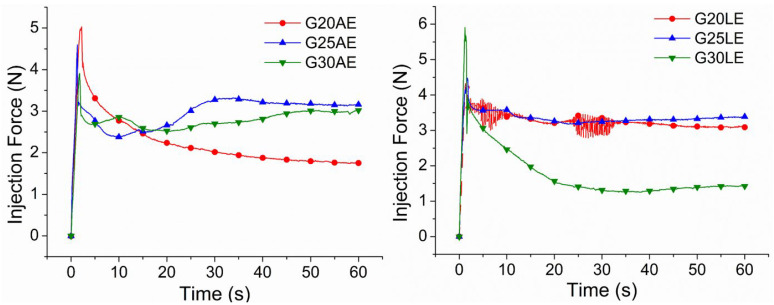
Force (N) vs. displacement time (s), representing the injectability properties of gels made with acetic acid (**left**) and lactic acid (**right**).

Gel formulations were examined for their simplicity of administration with a syringe. The representative compression force measurement as a function of gel injection time was observed and is shown in Figure 6. The initial sliding force (force required for the piston’s initial movement), dynamic sliding force (force required to maintain plunger movement), and maximum force (highest force observed during the experiment) needed to extrude the mixture were also measured and are listed in Table 3.

In Figure 6, changes in injection force were observed with increasing PEG concentration for all compositions. The compression force increased with the increase in PEG for gels obtained with acetic acid, but decreased in gels obtained with lactic acid. One possible explanation for this behavior is PEG’s function as a plasticizer, which increases the free volumes accessible between the CS chains, reducing the rigidity of its polymeric structure and enhancing segmental mobility [57,59].

Studies indicate that there are interactions between CS and the solvent (acid) in the solution, which are responsible for changing the CS properties. These interactions are influenced by the chemical structures of the acids, as well as the functional groups they carry [60,61]. Except for the G30AE and G30LE gels, when the influence of the acid type was observed in the gels, those prepared with lactic acid demonstrated a higher maximum force and sliding force than the gels made with acetic acid. Since the PEG concentration was the same in both systems, the difference in maximum and sliding force was caused by the steric effect of the various anions (acetate and lactate) on the CS [51,61].

Lactate ions are bulkier than acetate ions, resulting in increased rigidity of CS chains due to the difficulty in their organization during gel extrusion. In turn, acetate ions reduce the rigidity of CS chains by facilitating their organization and compaction when deformed [51,55]. Thus, raising the stiffness of CS chains leads to higher viscosity, resulting in an increase in their maximum and sliding forces. 

All gel formulations displayed compression force values below 30 N (recommended value as manual injection force limit), when the maximum limits of optimal injection force were considered [62]. Similar compressive force values have been reported by other researchers focused on the development of chitosan-based injectable biomaterials, such as scaffolds [63] and hydrogels [58]. According to these findings, all formulations can be employed as injectable systems.

### 2.7. In Vivo Hemostatic Efficacy of CS-PEG-EES Gels

**Figure 7:**
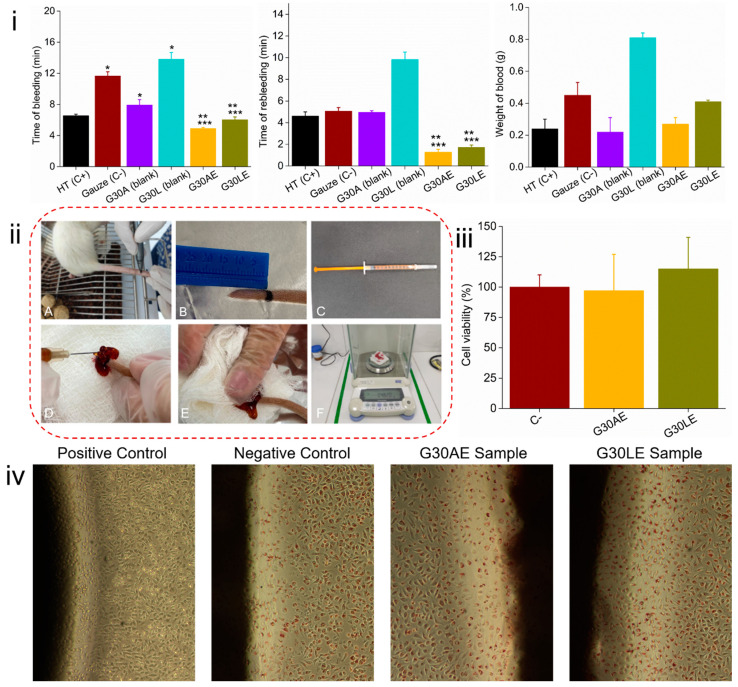
(**i**) Bleeding times, rebleeding times, and blood weight presented by the gels. Samples with (*) express a significant difference (*p* < 0.05) with the positive control Hemostank (HT); (**) with the Gaze negative control; and (***) with whites, according to the Kruskal-Wallis non-parametric test, followed by Dunn’s multiple comparisons tests. (**ii**) Images of the surgical procedure performed on the tails of Wistar rats. Demarcation of the surgical incision (A,B); Insulin syringe containing 1 mL of sample (C); Sample application at the incision site (D,E); Weighing the gauze to obtain the blood volume (F). (**iii**) Cytotoxicity of G30AE and G30LE gels by MTT assay. (**iv**) Photographs of the positive and negative controls, G30AE and G30LE compositions.

In this study, hemostatic activity was evaluated by quantifying the weight of blood and time of hemostasis for the induced bleeding in rats. The G30AE and G30LE formulations were chosen for hemostatic testing because of their superior rheological properties. Figure 7 illustrates the bleeding time, rebleeding time, and blood volume of the G30AE and G30LE gels, along with the blank (G30A and G30L), positive (HT), and negative (gauze) controls, and the surgical procedure.

Figure 7i reveals that the gels had a longer and significantly similar bleeding time in the blank groups, G30L (13 min and 49 s) and G30A (7 min and 55 s), followed by the negative control (11 min and 40 s). Shorter bleeding times were reported in the groups treated with EES-loaded gels, G30AE (4 min and 54 s) and G30LE (6 min and 2 s), which performed better than the Hemostank group (6 min and 34 s). However, they had no statistical differences (*p* > 0.05).

The rebleeding time was longer in the G30L group (9 min and 50 s), but there was no significant difference between it and the G30A groups, or the positive and negative controls (*p* > 0.05). The groups treated with ethanolic extract (EES), G30AE and G30LE, had shorter rebleeding times with a statistically significant difference compared with the blanks (G30A and G30L) and the negative control (*p* = 0.001). All groups had statistically similar blood weights (*p* > 0.05). The G30AE (0.22 g) group had the lowest blood weight, while the G30L (0.81 g) group had three times more blood than the G30AE and positive control—HT (0.24 g) groups.

Species of the genus *Jatropha* have been widely used in the treatment of illness and the subject of various research studies to demonstrate their chemical and therapeutic properties [64]. *Jatropha multifida* L. sap has been shown to accelerate clotting and healing time in wounded tissues. According to the findings, it is most effective during the inflammatory and proliferative phases of the healing process by activating growth factors and inducing wound re-epithelialization. Other studies indicate that the astringent effect of *J. multifida* sap improves hemostasis [65].

Other studies [66] have investigated the hemostatic activity and mechanism of action of *Jatropha gossypifolia* sap. Compared to the control group, the sap group demonstrated a 70-fold reduction in bleeding time in healthy adults and a 77-fold reduction in in vitro clotting time. Furthermore, a hemostasis mechanism for the sap was hypothesized, suggesting that it works by precipitating proteins, in this case, the components that participate in the coagulation cascade, leading to the synthesis of thrombin and lastly, the formation of a fibrin clot.

The chemical composition of *Jatropha mollissima* sap includes phenolic compounds, saponins, tannins, flavonoids, coumarins, alkaloids, and steroids. Furthermore, the sap contains a high concentration of polyphenols, which are compounds with antioxidant, hemostatic, and procoagulant properties, as well as tannins, which have antimicrobial, anti-inflammatory, healing, and hemostatic properties. These compounds act on bleeding by exerting a procoagulant [32] and astringent effect on the contraction of injured arteries and tissues, inducing platelet aggregation, plug formation, and clotting. They can also modify the activity of platelets, coagulation, and the fibrinolysis system of the endothelium, and have an inhibitory effect on thrombosis [17,28,33].

Catechin was shown to be one of the principal compounds in EES. It has been identified as a prominent ingredient of several plant extracts with strong hemostatic activity [67]. It is known to stimulate tissue regeneration by increasing collagen synthesis and blocking the formation of metalloproteinase enzymes [30]. Therefore, the presence of catechin in plants that show hemostatic activity and increased collagen synthesis may indicate a possible hemostatic action through the synergistic action of catechin with other compounds present in the EES.

We have previously investigated the hemostatic action of EES at doses of 25 and 40 mg·mL^−1^ [18], and found that the dose of 25 mg·mL^−1^ demonstrated the best outcomes, with a hemostasis time 1.72 times shorter than Monsel, a hemostatic medication already in use in the hemostasis treatment. In the present study, groups treated with EES (G130AE and G30LE) exhibited shorter bleeding times, lower blood volume, and less rebleeding compared with groups not treated with EES (G30A and G30L). These findings may be attributed to the EES’s hemostatic action, which works through the astringent effect due to the high amount of tannins [33], and protein precipitation, a process demonstrated by plants of the genus *Jatropha* [66,68].

The other components of the gels, in addition to the *J. mollissima* extract, possess hemostatic activity. PEG also stimulates the formation of clots that act as a mechanical seal, trapping blood [69]. Lactic acid binds to the proteins albumin and thrombin and induces the self-activation of coagulation factor XII, which can stimulate the production of insoluble blood clots [70]. Furthermore, lactic acid promotes re-epithelialization through enhanced water retention in the skin, stimulates fibroblast proliferation and collagen production, and acts on glycosaminoglycan synthesis [71].

CS is already known for its hemostatic and therapeutic effects. It promotes platelet aggregation and thrombus formation through a strong interaction between the positively charged protonated amine groups (–NH_3_^+^) and negatively charged platelet membrane [6]. When applied to wounds, CS gradually depolarizes, encouraging skin regeneration by promoting fibroblast proliferation, collagen, and hyaluronic acid deposition at the site [8,15]. Upon contact with blood, CS activates a gelation mechanism based on the formation of a three-dimensional network formed by the anchoring of the CS chain hydrophobes and membranes of blood cells [72].

Several studies have been conducted to explore the wound-healing ability of CS-PEG hydrogels [15]. The findings demonstrate that, although CS can interact with blood cells, it does not stop bleeding instantaneously. However, when combined with PPP micelles (PDLLA-PEG-PDLLA), faster in vitro coagulation and a more than 53% decrease in blood weight after treating rat liver hemorrhage were observed. As a result, groups treated with gel formulations containing CS and PEG demonstrated longer hemostasis and rebleeding periods than those treated with EES.

Aside from the hemostatic properties of its raw material, the gel also serves as a physical barrier at the injury site, as it promotes fast gelation and good adhesion to bleeding surfaces. As a result, it appears to be a highly valuable technique for attaining efficient hemostasis [8,15]. Thus, based on the findings, in formulations of gels made with CS and EES, particularly the G30AE formulation, the displayed hemostatic properties can be utilized as an effective hemostatic agent.

### 2.8. Cytotoxicity Experiments In Vitro

Cell viability testing is critical for any biomaterial utilized in therapeutic applications. Two preliminary techniques, Agar diffusion and MTT tests, were employed to assess the cytotoxicity of the extract gel samples G30AE and G30LE. These assays complement each other and ensure that the samples are not-cytotoxic and do not cause changes in cell viability. The results of both tests are shown in Figure 7.

The microscopically studied region of the positive control (toxic latex), negative control (Whatman paper filter number 1), G30AE, and G30LE samples are illustrated in Figure 7iv. The negative control and samples results revealed no bleaching cells around or under them (no halo formation was observed), indicating no cytotoxicity. However, bleaching of the cells was observed in the positive control. The bleaching zone around the positive sample was larger than 1 cm (Table S2, Supplementary Materials), resulting in a reactivity grade of 4. This preliminary assessment provides parameters for determining the presence or absence of cytotoxicity in the system under consideration.

According to ISO 10993-5:2009, a material is considered non-toxic if its cell viability surpasses 70% [73]. In consonance with the data in Figure 7iii, all samples displayed cell viability levels exceeding 70%. Furthermore, in the presence of the extracts, cells proliferated effectively, with the G30LE sample having a viability of 115% and stimulating cell proliferation. Acute toxicity studies carried out in rats indicate that *Jatropha mollissima* sap has low toxicity and its administration is safe [17]. Other studies also reveal that numerous chitosan biomaterials do not exhibit cytotoxicity towards L929 cells [74], even when loaded with *Jatropha mollissima* ethanolic extract [75]. Therefore, the gels are non-toxic, biocompatible, and suitable for use as biomaterials.

## 3. Materials and Methods

### 3.1. Chemicals

Sigma-Aldrich provided the high-molecular-weight CS (MW = 310–375 kDa; deacetylation degree = 75%), gallic acid ((OH)_3_C_6_H_2_CO_2_H, purity ≥ 98%), catechin (C_15_H_14_O_6_, purity ≥ 98%), vanillin (4-(HO)C_6_H_3_-3-(OCH_3_)CHO, purity ≥ 97%), and silica gel 60G (230–400 mesh, 0.04–0.06 mm) (San Luis, MO, USA). Ethyl alcohol P.A. (C_2_H_6_O, purity 98%), sulfuric acid P.A./ACS (H_2_SO_4_, purity 95–98%), phosphoric acid P.A. (H_3_PO_4_, purity 85%), hydrochloric acid P.A./ACS (HCl, purity 37%), dichloromethane P.A. (CH_2_Cl_2_, purity 99%), ethyl acetate P.A. (C_4_H_8_O_2_, purity 99%), PEG (PEG 400, H(OCH_2_CH_2_)_n_OH), and sodium carbonate (Na_2_CO_3_) were purchased from NEON (Suzano, SP, Brazil). Dinamica (Sao Paulo, Brazil) supplied the acetic acid P.A. (CH_3_CO_2_H, purity 99.8%). Lactic acid P.A. (C_3_H_6_O_3_, purity 85%) was purchased from Anidrol (Diadema, SP, Brazil). Silica slabs 60 F_254_ (0.015–0.040 mm) were purchased from Supelco (Bellefonte, PA, USA). HPLC grade acetonitrile (CH_3_CN, purity ≥ 99.9%) and Folin-Ciocalteu reagent were from Merck (Darmstadt, HE, Germany). J.T. Baker provided HPLC grade methanol (CH_3_OH, purity ≥ 99.9%) (Phillipsburg, NJ, USA). All solutions were made with 18.2 mΩ·cm ultrapure water obtained from a Merck Mili-Q Reference System (Darmstadt, HE, Germany). All of the chemical reagents utilized in this study were of analytical grade.

### 3.2. Ethics Committee

The study was approved by the Animal Research Ethics Committee of the CSTR: Center for Health and Rural Technology UFCG: Federal University of Campina Grande (CSTR/UFCG) under opinion No. 06/2020. The animals were researched and housed in accordance with the Rat Housing Guidelines in scientific institutions.

### 3.3. Jatropha mollissima Ethanolic Extract (EES) and Ethyl Acetate Fraction

*Jatropha mollissima* sap (Genetic Patrimony N° AD5B98B) was obtained in August 2019 from Boa Vista, Paraiba, Brazil, at geographic coordinates of 7°20′18.29″ S Long 36°14′17.11″ O. It was blended in a 1:1 ratio with ethyl alcohol and stored at ambient temperature (25 °C) for 72 h away from light. The solvent was then evaporated by rotary evaporation under a vacuum at 50 °C using an SL-126 rotary evaporator (SOLAB, Sao Paulo, Brazil). Finally, the solution was lyophilized after being frozen (−86 °C) in an ultra-freezer AmericanLab model 490L (Maringa, PR, Brazil) for 72 h. The resulting powdered ethanolic extract (EES) was stored in flasks in a dark place at room temperature.

An EES solution with a concentration of 0.16 g·mL^−1^ was made and transported to a separatory funnel where ethyl acetate was employed for liquid-liquid separation. The procedure was carried out by filling the funnel with 200 mL of ethyl acetate, and the liquid was gently swirled. It was necessary to wait until the phases were separated. The organic phase was separated from the aqueous phase and transferred to a flask. The procedure was repeated twice more. Finally, the Ethyl Acetate Fraction (EAF) was concentrated by rotary evaporation at 50 °C under vacuum, weighed, and kept away from light. The yield of EAF was 0.1371 g (equivalent to 0.083% of the EES mass used).

### 3.4. Synthesizing CS-PEG-EES Gels

The gel formulations were made by dissolving 0.5 g of CS in aqueous solutions of acetic acid at 1% (*v*/*v*) and lactic acid at 2% (*v*/*v*), for 1 h at 25 °C with mechanical stirring at 380 rpm. PEG and EES were then added to the solution and stirred for another hour under the same conditions. The resulting gel was transferred to a falcon tube and stored in the dark at 4 °C. To eliminate bubbles, the gels were centrifuged at 3500 rpm for 5 min in a refrigerated centrifuge Novatecnica NT 835 (Piracicaba, SP, Brazil). The gel formulations were made with a final volume of 50 mL. The amounts of reagents used in the gels are presented in Table 4.

**Figure 8:**
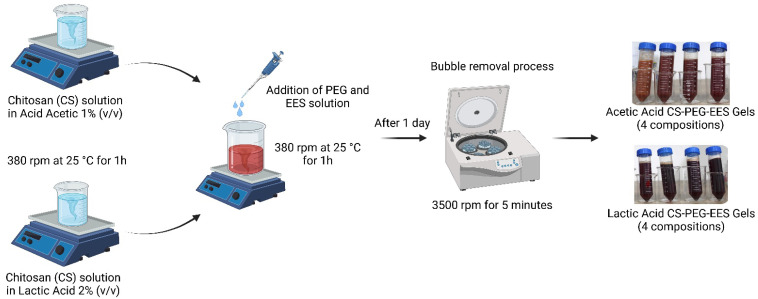
Methodology for obtaining CS-PEG-EES gels.

**Figure 9:**
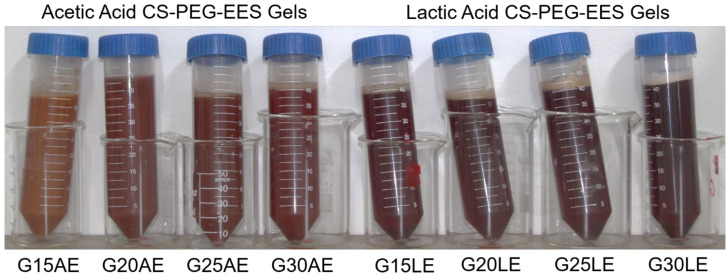
Illustration of the CS-PEG-EES gels obtained with acetic and lactic acids.

The conditions for preparing the gels were established based on previous research. Initially, formulations containing 2.5 and 5 mL of PEG were employed but they resulted in low-viscosity gels that were not further investigated. Lactic acid was chosen for its hemostatic and healing capabilities and its lack of odor. However, since lactic acid aqueous solution at 1% (*v*/*v*) did not completely dissolve CS in our experiments, the 2% concentration was chosen. Based on our previous findings, the EES was used at a concentration of 25 mg·mL^−1^ [18]. Figure 8 depicts the procedure for preparing CS-PEG-EES gels, while Figure 9 illustrates the gels.

### 3.5. Phytochemical Analysis

#### 3.5.1. Thin-Layer Chromatography (TLC)

The thin-layer chromatography (TLC) technique was employed to identify potentially isolated substances. Analyses were performed on 60G silica gel plates (2 × 5 cm) coated with an F_254_ indicator as the stationary phase. A mobile phase consisting of a mixture of dichloromethane:acetone (3:7, *v*/*v*) was used for EES fingerprinting, while dichloromethane:ethyl acetate (7:3, *v*/*v*) was used to verify the isolation of substances from the EAF. After obtaining the chromatograms, the plates were dried and the compounds were revealed under a UV illumination lamp (254 nm) and by using phosphoric vanillin.

#### 3.5.2. Fractionation of the Ethyl Acetate Fraction

To purify the EAF, column chromatography (CC) was used. A 10 × 100 mm glass column filled with a stationary phase of silica gel (230–400 mesh, 0.04–0.06 mm) was utilized for this purpose, with a sample mass-to-silica ratio of 5% (*w*/*w*). The mobile phase was composed of dichloromethane and ethyl acetate in ascending polarity order, beginning with 100% dichloromethane and increasing the amount of ethyl acetate by 10% during the separation. Finally, the eluent was collected in appropriately labeled containers. TLC was used to examine the chromatographic profile of the compounds in the containers after the solvent was evaporated. Magnetic resonance spectroscopy, infrared spectroscopy, mass spectrometry, and high-performance liquid chromatography were employed to evaluate the extracted substances.

#### 3.5.3. Nuclear Magnetic Resonance (NMR)

Nuclear magnetic resonance (NMR) tests were performed using a Bruker Avance Neo 500 Spectrum (Bruker, Billerica, MA, USA) operating at 500 MHz for the acquisition of the ^1^H spectrum and 125 MHz for the ^13^C spectrum. This analysis was conducted to identify the chemical structure of the constituent obtained from EAF. To obtain the ^1^H and ^13^C spectra, dimethylsulfoxide-d_6_ was used as a solvent and the following parameters were employed: temperature of 25 °C, number of scans: 64, receiver gain: auto, and acquisition time: 2 min. The acquired data were processed using the Mnova Version 14.2.3 program.

#### 3.5.4. Mass Spectrometry (MS) by Direct Infusion

An AxION 2 TOF mass spectrometer (Perkin Elmer, Waltham, MA, USA) was used to discover the molecular mass of purified compounds from EAF. The analyses were conducted in negative electrospray ionization (ESI) mode. The sample was prepared by diluting it in methanol to a concentration of 10 µg·mL^−1^ and was then infused in direct infusion mode at a flow rate of 10 µL·min^−1^ using a 1 mL Hamilton syringe and an infusion pump integrated into the equipment. To ensure the accuracy of the analysis, the equipment was calibrated with the standard solution ESI Tuning Mix Agilent Technologies (Santa Clara, CA, USA). The spectra were acquired in *full scan* mode in the range of 50–650 *m*/*z* at the rate of 1 spectrum per second. Nitrogen was used as a nebulizer gas at a flow rate of 10 mL·min^−1^ at a temperature of 350 °C and a pressure of 80 psi. The spectrum was recorded until its amplitude remained constant.

#### 3.5.5. High-Performance Liquid Chromatography Coupled to Diode Array Detector (HPLC–DAD)

To obtain the chromatographic profile (fingerprint) of the EES and identify one of its major substances, the HPLC–DAD technique was applied. The chromatographic analyses were performed on a liquid chromatography system (Perkin Elmer, Waltham, MA, USA) equipped with an automatic injector, oven, and pump coupled to a diode array detector (DAD) Flexar PDA Plus Detector. All solvents used were HPLC grade, filtered (0.45 µm), and ultrasonically degassed.

A chromatographic method was developed by testing various chromatographic conditions, including different solvents and proportions, oven and column temperatures, and, at the end, the analyses were performed considering the best parameters. The eluents used were a 1% (*v*/*v*) aqueous acetic acid solution (A) and acetonitrile (B) on a C18 column C18 column (250 × 4.6 mm, 5 µm particle size). The analyses were conducted in gradient mode under the following conditions: 5% B, 0–1 min; 5–10% B, 1–5 min; 10–20% B, 5–14 min; 20–21% B, 14–24 min; 21–95% B, 24–29 min. 5% B was used for 5 min as an equilibration step and 95% B for 10 min as a wash step. The total analysis time was 29 min. The flow of eluents was maintained at 0.48 mL·min^−1^ and 20 µL of the sample was injected. The oven temperature was maintained at 30 ± 1 °C.

The retention time and ultraviolet spectra of the substances were monitored at a wavelength of 280 nm, with UV-Vis spectrum data acquired in the range of 200–400 nm. EES samples were prepared at a concentration of 2.5 mg·mL^−1^ in a 95:5 water:acetonitrile (*v*/*v*) solution and filtered with 0.45 µm membranes. This proportion was chosen because preparing samples in liquid chromatography in the same initial proportion of the mobile phase provides a better separation of the substances.

#### 3.5.6. Determination of Phenolic Contents

The total polyphenols in the ethanolic extract of *J. mollissima* were determined using the Folin-Ciocalteu method [76]. Initially, an aqueous stock solution of gallic acid (100 µg·mL^−1^) and its dilutions in concentrations ranging from 1.0 to 40.0 µg·mL^−1^ were prepared. Subsequently, 250 µL of each dilution was mixed with 2.5 mL of 2 M Folin-Ciocalteu reagent and 2 mL of 20% (*w*/*v*) sodium carbonate solution. After 30 min, the readings were conducted in a spectrophotometer model UV-1800 (Shimadzu, Kyoto, Japan) at room temperature (25 °C) using a quartz cuvette with 1 cm optical path model Q-204, with the absorbance values of the wavelength at 770 nm recorded. 

For the EES sample, 1 mg of EES was solubilized in 10 mL of ethyl alcohol to obtain a concentration of 100 μg·mL^−1^. The other preparation steps were conducted in a similar manner to that of the gallic acid solution. The concentration of total polyphenols was expressed as a function of the equivalent concentration of gallic acid (mg GAE/g) of the dry extract. All measurements were performed in triplicate. The phenolic content was calculated using the formula:

TPC = C × (*V*/*M*), (1)

where:

TPC: total phenolic content in mg GAE/g of the dry extract;
C: gallic acid concentration in μg·mL^−1^ obtained for the EES sample through the calibration curve;
*V*: volume in mL of solvent used in preparing the EES sample;
*M*: EES mass in mg used in sample preparation.

#### 3.5.7. Determination of Condensed Tannins Contents

To determine the total concentration of condensed tannins, the vanillin-hydrochloric acid (HCl) method was used [77]. First, a methanolic stock solution of catechin (550 µg·mL^−1^) and its respective dilutions at concentrations of 10–550 µg·mL^−1^ were prepared. Subsequently, 3 mL of a 4% (*w*/*v*) vanillin solution, 500 µL of each of the diluted solutions, and 1.5 mL of a 0.75 M HCl solution were transferred to test tubes and immersed in water at 22 °C for 20 min. The readings were taken in a spectrophotometer model UV-1800 (Shimadzu, Kyoto, Japan) at room temperature (25 °C) using a quartz cuvette with an optical path of 1 cm model Q-204. 

For sample preparation, 10 mg of EES were solubilized in 10 mL of ethanol to obtain an EES solution at a concentration of 1000 μg·mL^−1^. Next, the sample preparation was conducted in a similar manner to that of the catechin stock solution. The absorbance values of the wavelength at 500 nm were recorded. The concentration of total condensed tannins was expressed as the equivalent concentration of catechin (mg CE/g) of the dry extract. All measurements were performed in triplicate. The condensed tannin content was calculated using the formula:

TTC = C × (*V*/*M*) (2)

where:

TTC: total tannin content in mg EC/g of the dry extract;
C: catechin concentration in μg·mL^−1^ obtained for the EES sample through the calibration curve;
*V*: volume in mL of solvent used in preparing the EES sample;
*M*: EES mass in mg used in sample preparation.

### 3.6. Gels Characterizations

#### 3.6.1. Fourier Transform Infrared Spectroscopy (FTIR)

FTIR analysis was used to investigate the interactions between the gel components, to identify EES incorporation into gels and establish connections with their physicochemical properties. FTIR was also applied to identify the compound obtained from EAF fraction. The spectra were acquired on a Spectrum 400 Spectrometer (Perkin Elmer, Waltham, MA, USA) coupled with an attenuated total reflectance (ATR) accessory equipped with a thallium bromoiodide (KRS-5) crystal over a range of 4000–600 cm^−1^, 32 scans and with a resolution of 4 cm^−1^. Prior to data collection, the gels were dried at 40 °C for 24 h.

#### 3.6.2. Rheological Analysis

The rheological properties of the gels were evaluated using a HAAKE™ MARS™ rheometer (ThermoFisher Scientific, Waltham, MA, USA), equipped with a P35Til rotor and plate-plate geometry. The samples were analyzed at constant temperatures of 25 °C and 37 °C. For all temperature conditions, a shear rate sweep ranging from 0.001 to 0.1 was used with a fixed frequency of 1.592 Hz for 60 s and a gap of 0.5 mm in all temperature ranges.

#### 3.6.3. Thermal Stability

Thermal stability tests were carried out to measure the stability of the gels in a temperature range while maintaining a constant frequency and shear rate. The test was conducted on a HAAKE™ MARS™ plate rheometer (ThermoFisher Scientific, Waltham, MA, USA), equipped with a P35Til rotor and plate-to-plate geometry working with a gap value of 0.5 mm and a fixed frequency of 1.592 Hz. The temperature range was 20–40 °C.

#### 3.6.4. Injectability

The compressive force and injection profile of the CS-PEG gels were determined on an Instron 3366 universal mechanical testing machine (Instron, Norwood, MA, USA). For the test, a 5 mL syringe (Descarpack, São Paulo, SP, Brazil), without a needle, was filled with 2 mL of gel and fixed vertically under the plate of the traction bench set in compression mode. A constant compressive force of 10 mm·min^−1^ was applied to the syringe at a constant speed and removed after 1 min. The compression force (N) was recorded as a function of compression length (mm). The test was zzzconducted at room temperature (25 °C).

#### 3.6.5. Cytotoxicity—MTT

To verify the cytotoxicity of the best gel compositions, cell compatibility was evaluated according to the direct contact method, and samples were prepared using the methodology to prepare liquid extracts, following the methodologies proposed by the ISO 10993-5 [73] and ISO 10993-12 [78]. Briefly, 0.2 g of the G30AE and G30LE compositions were weighed and sterilized for 30 min by UV radiation in a laminar flow cabinet. Then, the gels were transferred to sterile 20 mL vials, and 1 mL of the extraction medium (10% fetal bovine serum (FBS) + 90% RPMI 1640 medium) was added. The samples were then incubated at 37 °C for 24 h and after incubation, and extract samples were collected.

L929 cells (ATCC NCTC clone 929) were cultured in RPMI 1640 medium (Invitrogen Corporation, Waltham, MA, USA) containing 10% FBS and 1% antibiotic-antimycotic (Invitrogen Corporation, Waltham, MA, USA) in culture flasks and incubated in a humidified oven at 37 ± 1 °C with an atmosphere of 5 ± 1% CO_2_. The cells were trypsinized and adjusted to a concentration of 1.1 × 10^5^ viable cells per mL using an Invitrogen automatic cell counter (ThermoFisher Scientific, Waltham, MA, USA), and then seeded on 96–well culture plates for 24 h. After cultivation, 50 µL of the medium extracted from the samples was added to each of the wells and the cells were incubated for another 24 h. Finally, the cytotoxicity assay was performed using the MTT method.

After the incubation time, the culture medium of each well was removed, and 100 µL of MTT solution (5 mg·mL^−1^) was added and incubated for 4 h. Then, 100 µL of DMSO was added to dissolve the formazan crystals, and the absorbance of the wells was measured at wavelengths of 570 and 620 nm using a Victor X3 Spectrophotometer (Perkin Elmer, Waltham, MA, USA) for the viability calculation.

#### 3.6.6. Cytotoxicity—Agar Diffusion Method

The cytotoxicity of the G30AE and G30LE gels was also evaluated by the agar diffusion method. The samples were prepared using the same methodology to prepare liquid extracts as that used for the MTT test. After preparing the sample extracts, a filter paper measuring 100 mm^2^ was soaked in the extracts and sterilized for 1 h by UV radiation in a laminar flow cabinet. Then, positive controls (Latex for tourniquet) and negative controls (Whatman Filter Paper Number 1) were prepared.

A suspension of L929 cells with a concentration of 1.3 × 10^5^ viable cells per mL in RPMI 1640 medium supplemented with 10% fetal bovine serum (FBS) was used for the assay. 4 mL of the cell suspension were collected in each hole of the plastic culture microplate (3.5 cm in diameter). Then, duplicate cultures were prepared for the sample, negative control, positive control, and blank. The cultures were incubated in a humidified oven at 37 ± 1 °C with 5 ± 1% CO_2_ for 48 h. After incubation, cultures that showed a uniform cell monolayer close to confluence (greater than 80%) were used for the assay. Thus, the culture medium of the microplates was aspirated, and the monolayer of each hole in the plate was washed with 2 mL of PBS solution, which was then also aspirated. Soon after, 1 mL of the covering medium composed of 1.8% agar, 0.01% neutral red dye, and 2-fold concentrated MEM was added to each well in equal amounts.

The plates were left in the laminar flow hood for 10 min to allow the agar to solidify at room temperature (25 °C). Samples, negative control, and positive control were placed in contact with the solidified surface of the agar, in the center of each plate, in duplicate cultures. The plates were incubated in an inverted position for 24 h in a humidified oven at 37 ± 1 °C, with 5 ± 1% CO_2_, and wrapped in aluminum foil to avoid cell damage by photoactivation of neutral red. Cell discoloration was determined using a light inverted microscope model TS100 (Nikon, Tokyo, Japan).

#### 3.6.7. In Vivo Hemostatic Activity Assay

To determine the hemostatic properties of the gels, an animal study was authorized by the ethics committee of the Federal University of Campina Grande (protocol No. 06/2020) and conducted based on the methodology proposed by Sogut, Erdogan, Kose, Boleken, Kaya, Gokdemir, Ozgonul, Iynen, Albayrak and Dokuzoglu [79]. The tail region was chosen for the hemostatic test because it is highly vascularized, has a prominent central artery, two main lateral veins and a dense network of smaller blood vessels and the presence of arteriovenous anastomosis [80]. Briefly, 36 adult male Wistar rats (~300 g) were divided into six groups (*n* = 6): Groups G30AE, G30LE, G30A (white), G30L (white), positive control—Hemostank (HT), negative control (gauze) and anesthetized. Then, the surgical area was antisepsis and a 10-mm distal segment of the tail tip was transversally amputated with a scalpel blade. After amputation, the lesion was treated with 1 mL of the transferred gel using a 1 mL insulin syringe attached to a 25 × 7 mm needle with continuous application for 20 s, followed by gentle compression with sterile gauze for 1 min to ensure the gel remained in contact with the wound. A chronometer was used to measure the time taken to reach hemostasis. The time counting began at the moment of incision and ended when bleeding stopped completely. The amount of blood and the time of bleeding and rebleeding were recorded.

To determine the blood weight, the sterile gauze was weighed before and after the procedure. The blood weight (g) was obtained using the formula:

P_(S)_ = G_(1)_ − G_(0)_ (3)

where:

P_(S)_: Weight of blood;
G_(1)_: Weight of gauze + 1 mL of gel + blood;
G_(0)_: Weight of gauze + 1 mL of gel.

Each animal was monitored for 20 min, even if the bleeding stopped, to verify the occurrence of possible rebleeding.

After the surgical procedure, animals were intraperitoneally sedated with ketamine hydrochloride—Ketamine^®^ (Vetecia, Jararei, SP, Brazil) (100 mg·kg^−1^) and xylazine hydrochloride—Calmiun^®^ (União Química, Sao Paulo, SP, Brazil) (10 mg·kg^−1^) intraperitoneally. Then, the rats were euthanized by the cervical dislocation technique [23], which was performed according to the AVMA Guidelines for the Euthanasia of Animals: 2013 Edition [24]. Euthanasia was confirmed by the absence of eyelid reflex, thoracic movements and heartbeats for three minutes. Euthanasia was carried out to prevent inflammation of the tail, bacterial contamination, and possible transmission of diseases to healthy rats. Additionally, the caudal segment of the animal was removed, which is considered irreversible damage and therefore subject to euthanasia [24]. After euthanasia, the bodies of the animals were incinerated.

### 3.7. Statistical Analysis

In vivo Hemostatic Activity Assay: Kolmogorov–Smirnov and Levene’s tests were used to select the appropriate statistical method based on the distribution model and data variance. Since the results of the hemostatic analysis did not follow a normal distribution, the non-parametric Kruskal-Wallis test was used, followed by the Dunn test to determine differences between groups (*p* < 0.05). GraphPad Prism software, version 5.0 (GraphPad Software Inc., San Diego, CA, USA) was used to analyze the data.

Cytotoxicity assays (MTT): The Grubbs Test for Outliers was applied to evaluate the results of the cell viability calculation, with a significance level set at *p* < 0.05. Data were analyzed using Action Stat software version 3.6.331 (EstatCamp, São Carlos, SP, Brazil).

## 4. Conclusions

The physicochemical, hemostatic, and cytotoxic properties of chitosan-polyethylene glycol (CS-PEG) gels loaded with ethanolic extract (EES) of *Jatropha mollissima* were investigated in this work. Interactions between the gel components were detected by the formation of hydrogen bonds between the –NH_2_ groups present in CS and the ether groups (C–O–C) in PEG and by the existence of EES characteristic bands in the gel spectra. The gels were demonstrated to be easily injectable and stable at both room and human body temperature. In vivo hemostasis assays showed that the gels induced a hemostatic effect, resulting in lower bleeding time and volume compared with the control group and Hemostank. These findings may be attributed to the procoagulant, protein precipitation, and the astringent effect of *Jatropha mollissima* EES due to its high polyphenols and tannins concentration. Finally, the gels demonstrated adequate cellular biocompatibility, with no toxicity to fibroblasts (L929). Based on these results, CS-PEG-EES gels, especially the formulation prepared with acetic acid, are prime candidates for hemorrhage control. The next steps will involve a phytochemical investigation of *Jatropha mollissima* sap, as well as in vivo studies to evaluate the healing and biodegradation properties of the CS-PEG-EES gels. Additionally, hemolysis, prothrombin time, and viscoelastic hemostatic assays will be conducted to further support the findings reported in this study.

## Figures and Tables

**Table 1:** ^1^H and ^13^C NMR spectra assignments of fraction A9 (in DMSO-d_6_ at 25 °C) and catechin according to [20,21,22,23].

Position	Fraction A9, DMSO-d_6_		Literature–Catechin, DMSO-d_6_	
	δ_C_	δ_H_	δ_C_	δ_H_
2	81.42	4.47 (1H, d, J = 7.2 Hz)	81.1	4.46 (d)
3	66.74	3.80 (1H, m)	66.4	3.82 (m)
4	28.26	2.64 (Ha) (1H, dd, J = 16.0; 5.2 Hz); 2.34 (Hb) (1H, dd, J = 16; 8.0 Hz)	28.0	2.66 (dd); 2.37 (dd)
5	156.62	-	156.3	-
6	95.58	5.87 (1H, d, J = 2.3 Hz)	95.2	5.88 (d)
7	156.88	-	156.6	-
8	94.31	5.68 (1H, d, J = 2.3 Hz)	94.0	5.68 (d)
9	155.79	-	155.5	-
10	99.54	-	99.2	-
1′	131.02	-	130.7	-
2′	114.94	6.70 (1H, d, J = 2.3 Hz)	114.6	6.72 (d)
3′	144.88	-	145.0	-
4′	145.29	-	145.0	-
5′	115.53	6.67 (1H, d, J = 8.0 Hz)	115.2	6.69 (d)
6′	118.90	6.58 (1H, dd, J = 8.0; 2.3 Hz)	118.6	6.60 (d)
3-OH	-	4.90 (1H, d, J = 5.2 Hz)	-	4.86 (d)
5-OH	-	9.15 (1H, s)	-	9.18 (s)
7-OH	-	8.91 (1H, s)	-	8.94 (s)
3′-OH	-	8.79 (1H, s)	-	8.86 (s)
4′-OH	-	8.84 (1H, s)	-	8.82 (s)

**Table 2:** Total phenolic and condensed tannins content present in the *J. mollissima* ethanolic extract (*n* = 3).

Sample	TPC (mg GAE/g Dry Extract)	TTC (mg CE/g Dry Extract)
EES	242.84 ± 0.90	512.30 ± 6.83

TPC: total phenolic content; TTC: total tannin content.

**Table 3:** Data on injectability of gel formulations.

Sample	Initial Glide Force (N)	Dynamic Glide Force (N)	Maximum Force (N)
G20AE	5.02 ± 0.04	1.75 ± 0.02	1.79 ± 0.02
G20LE	4.32 ± 0.10	3.11 ± 0.01	3.12 ± 0.02
G25AE	4.59 ± 0.37	3.19 ± 0.01	3.32 ± 0.01
G25LE	4.48 ± 0.04	3.30 ± 0.01	3.39 ± 0.02
G30AE	3.90 ± 0.11	2.98 ± 0.01	3.02 ± 0.01
G30LE	5.90 ± 0.23	1.37 ± 0.01	1.43 ± 0.01

**Table 4:** List of CS-PEG gel formulations.

Compositions	Raw Materials				
	Acetic Acid (*v*/*v*)	Lactic Acid (*v*/*v*)	PEG 400 (*v*/*v*)	Chitosan (*w*/*v*)	EES (mg·mL^−1^)
G15AE	1%	–	15%	1%	25
G20AE	1%	–	20%	1%	25
G25AE	1%	–	25%	1%	25
G30AE	1%	–	30%	1%	25
G15LE	–	2%	15%	1%	25
G20LE	–	2%	20%	1%	25
G25LE	–	2%	25%	1%	25
G30LE	–	2%	30%	1%	25

EES = Ethanolic Extract of *J. mollissima*.

## Data Availability

Not applicable.

## Supplementary Materials

The following supporting information can be downloaded at: https://www.mdpi.com/article/10.3390/ph16101399/s1, Figure S1: Thin layer chromatography (TLC) plates of fraction A9 from ethyl acetate fraction (EAF). TLC plates revealed by UV (254 nm) (a) and Phosphoric Vanillin plus heating (b). Mobile phase: 7:3 (Dichloromethane:Ethyl Acetate); Figure S2: Mass spectrum obtained by ESI in negative mode of fraction A9 from EAF; Figure S3: NMR 1H spectrum of fraction A9 from EAF in dmso-d6 at 25 °C; Figure S4: NMR 13C spectrum of fraction A9 from EAF in dmso-d6 at 25 °C; Figure S5: FTIR spectrum of fraction A9 from EAF; Figure S6: Calibration curve obtained with the gallic acid standard for quantification of total polyphenols by UV-VIS spectroscopy. Detection at 770 nm; Figure S7: Calibration curve obtained with the catechin standard for quantification of total condensed tannins by UV-VIS spectroscopy. Detection at 500 nm; Figure S8: Elastic (G′) and plastic (G″) loss modulus of CS-PEG-EES gels prepared with acetic acid at 25 °C; Figure S9: Elastic (G′) and plastic (G″) loss modulus of CS-PEG-EES gels prepared with lactic acid at 25 °C; Figure S10: Elastic (G′) and plastic (G″) loss modulus of CS-PEG-EES gels prepared with acetic acid at 37 °C; Figure S11: Elastic (G′) and plastic (G″) loss modulus of CS-PEG-EES gels prepared with lactic acid at 37 °C; Table S1: Catechin purity data determined by calculating its relative area on HPLC–DAD chromatogram; Table S2: Cytotoxicity classification of the G30AE, G30LE, negative and positive controls obtained by agar diffusion test.
