# Supplementary material for: Chitosan-PEG Gels Loaded with Jatropha mollissima (Pohl) Baill. Ethanolic Extract: An Efficient and Effective Biomaterial in Hemorrhage Control
Source: Pharmaceuticals (Basel). 2023 Oct 3;16(10):1399. doi: 10.3390/ph16101399 (PMC10609772; doi:10.3390/ph16101399)
Supplement: Supplementary file 1 [file pharmaceuticals-16-01399-s001.zip › pharmaceuticals-2280621-supplementary.pdf]

Physicochemical, toxicologic and hemostatic properties of chitosan-PEG gels loaded with *Jatropha mollissima* (Pohl) Baill. ethanolic extract

Rodrigues, J. F. B.[1], Queiroz, J. V. S.[1], Medeiros, R. P.[1], Santos, O. R.[1], Fialho, D. A.[1], Neto, J. E. S.[1], Santos, R. L.[2], Barbosa, R. C.[1], Sousa, W. J. B.[1], Medeiros, L. A. D. M.[1], Silva, S. M. L.[1], Montazerian, M.[1], Fook, M. V. L.[1], Amoah, S. K. S.[1]

[1]Northeast Laboratory for Evaluation and Development of Biomaterials, Academic Unit of Materials Science and Engineering, Federal University of Campina Grande, Campina Grande, 58429-000, Paraiba, Brazil.
[2]Federal University of Juiz de Fora, Life Science Institute, Department of Dentistry, Governador Valadares, 36036-900, Minas Gerais, Brazil.

Supplementary Material

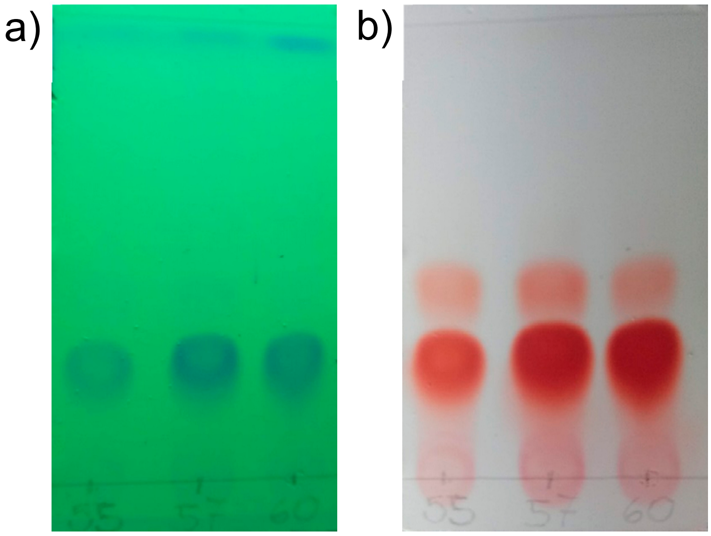

Figure S1.: Thin layer chromatography (TLC) plates of fraction A9 from ethyl acetate fraction (EAF). TLC plates revealed by UV (254 nm) (a) and Phosphoric Vanillin plus heating (b). Mobile phase: 7:3 (Dichloromethane:Ethyl Acetate).

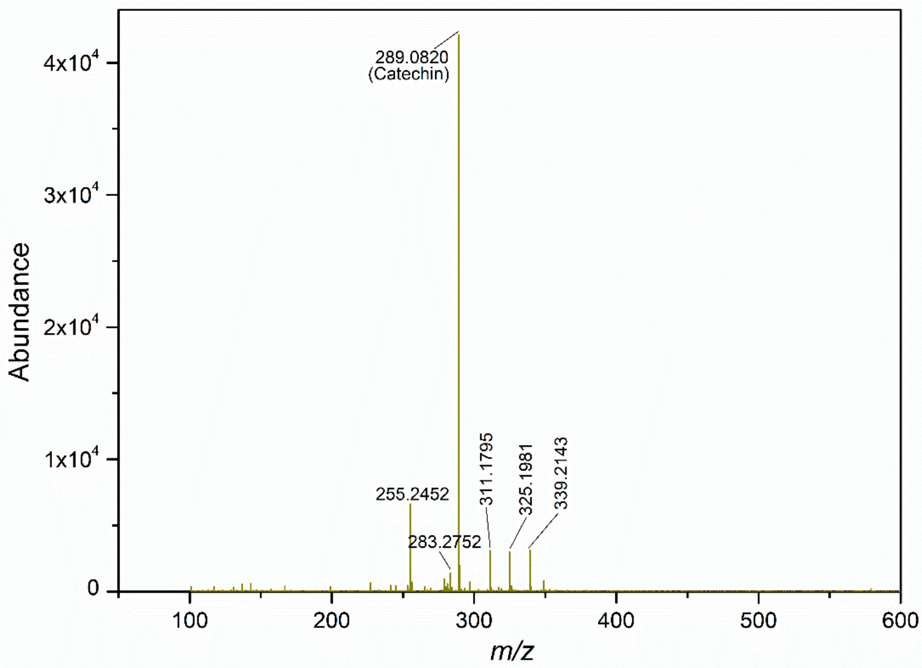

Figure S2.: Mass spectrum obtained by ESI in negative mode of fraction A9 from EAF.

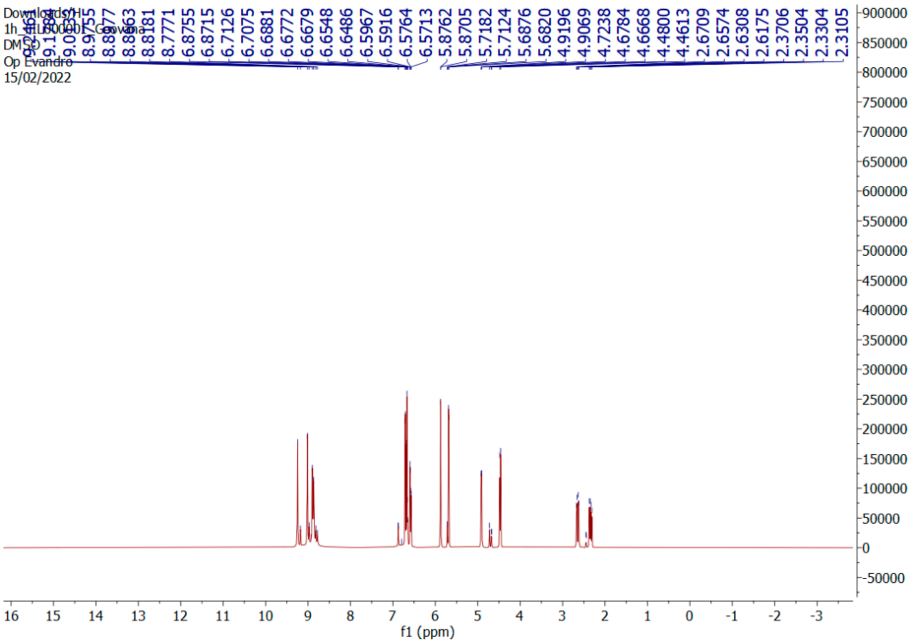

Figure S3.: NMR ^{1}H spectrum of fraction A9 from EAF in dmso-d_6 at 25 °C.

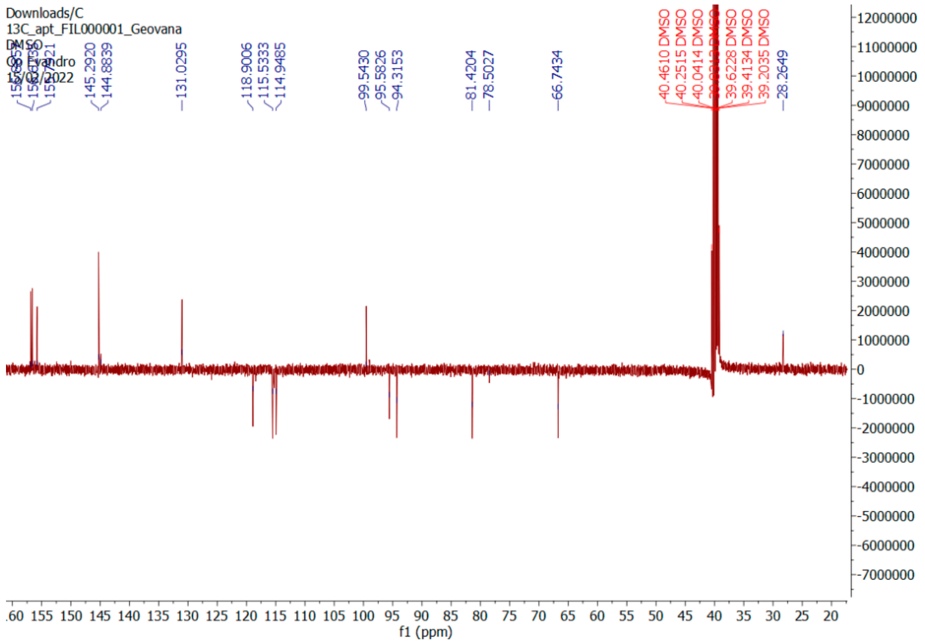

Figure S4.: NMR ^{13}C spectrum of fraction A9 from EAF in dmso-d_6 at 25 °C.

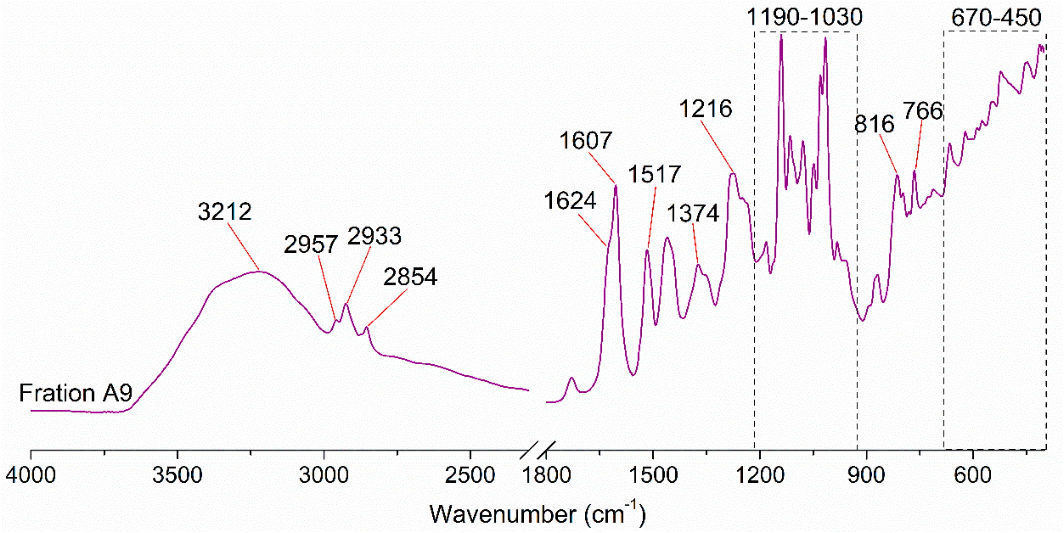

Figure S5.: FTIR spectrum of fraction A9 from EAF.

Table S1.: Catechin purity data determined by calculating its relative area on HPLC–DAD chromatogram.

Peak	Retention Time (RT)	Relative Area (%)	Purity (%)
**1**	17.82	1.48	-
**2**	18.67	5.12	-
**3**	19.58	1.90	-
**4***	20.18	79.42	79.42
**5**	23.13	12.08	-

*Peak 4 – Catechin.

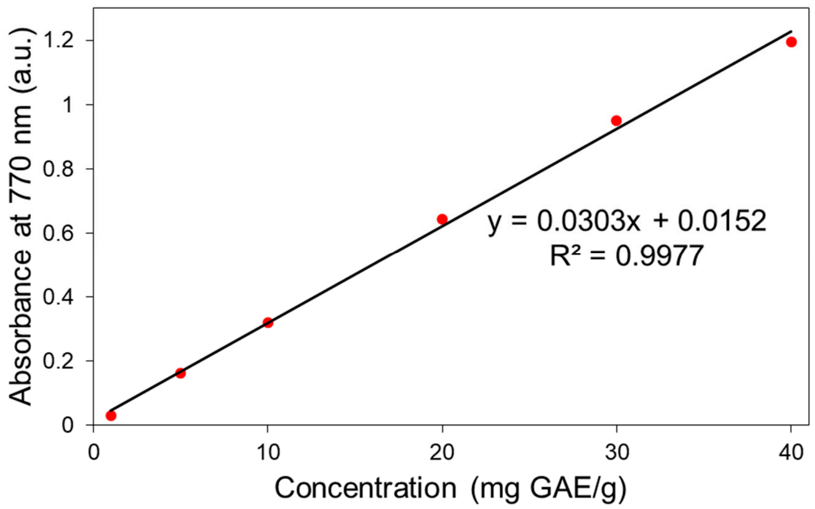

Figure S6.: Calibration curve obtained with the gallic acid standard for quantification of total polyphenols by UV-VIS spectroscopy. Detection at 770 nm.

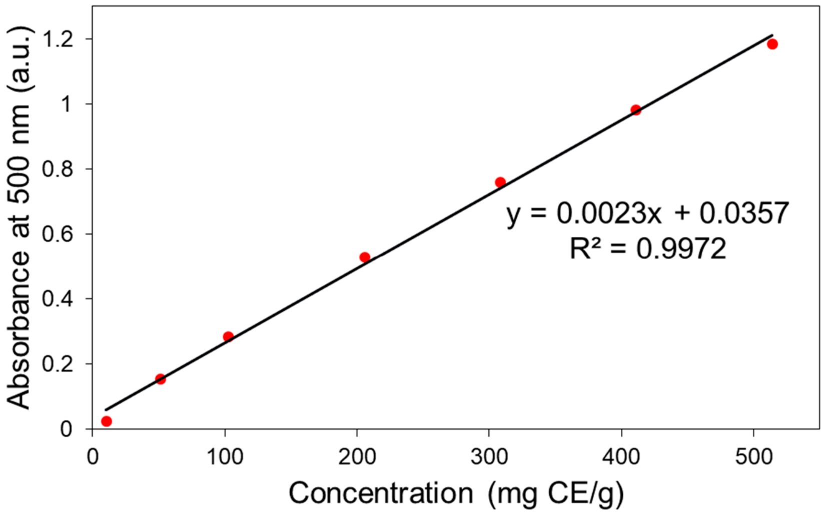

Figure S7.: Calibration curve obtained with the catechin standard for quantification of total condensed tannins by UV-VIS spectroscopy. Detection at 500 nm.

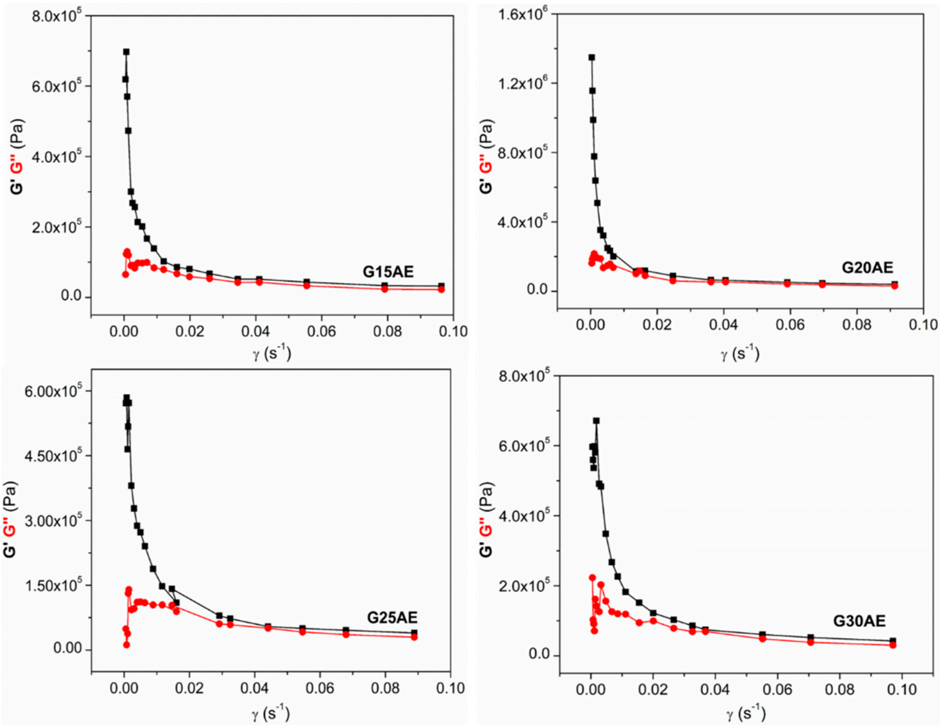

Figure S8.: Elastic (G') and plastic (G'') loss modulus of CS-PEG-EES gels prepared with acetic acid at 25 °C.

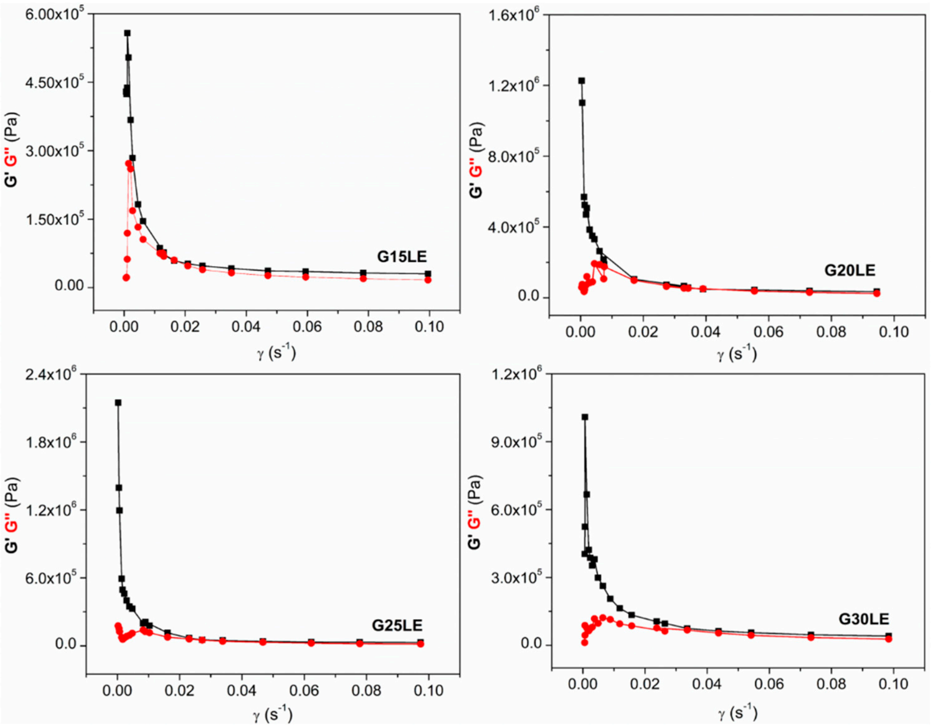

Figure S9.: Elastic (G') and plastic (G'') loss modulus of CS-PEG-EES gels prepared with lactic acid at 25 °C.

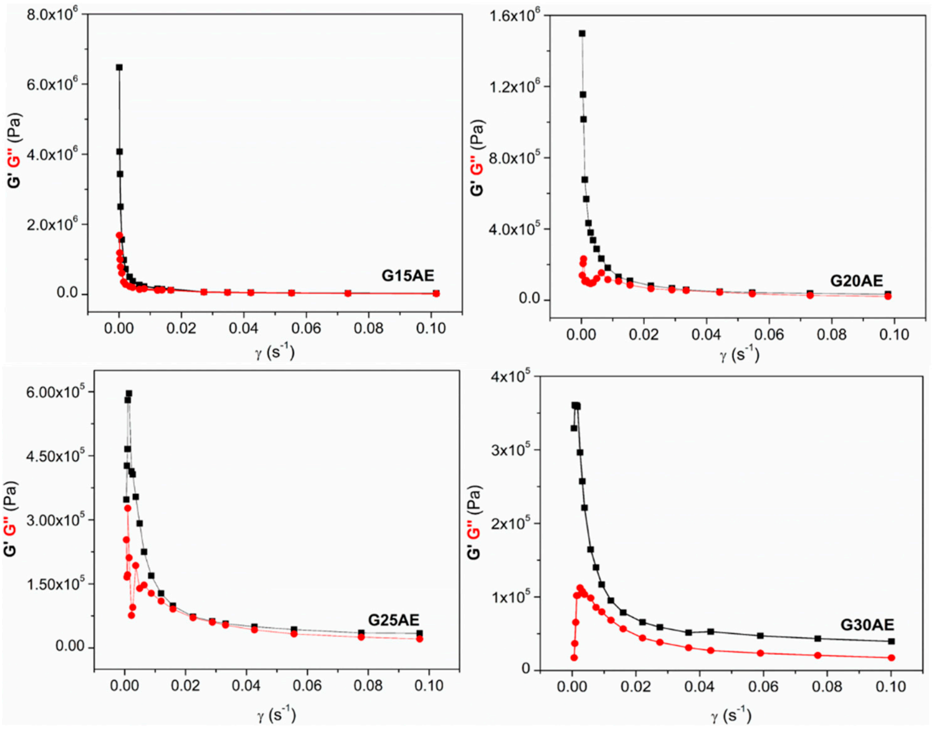

Figure S10.: Elastic (G') and plastic (G'') loss modulus of CS-PEG-EES gels prepared with acetic acid at 37 °C.

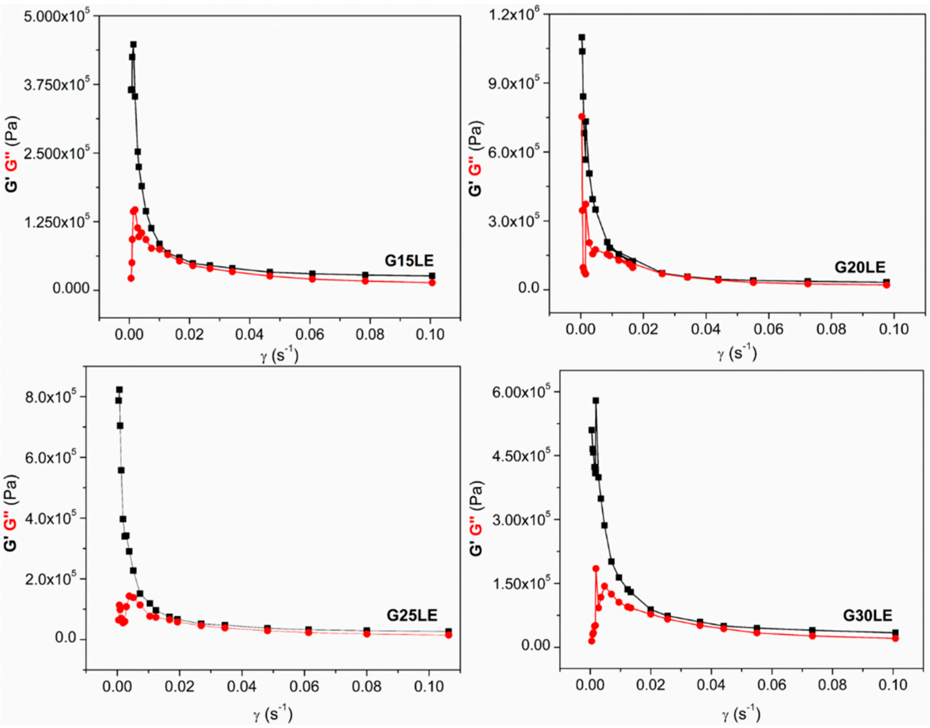

Figure S11.: Elastic (G') and plastic (G'') loss modulus of CS-PEG-EES gels prepared with lactic acid at 37 °C.

Table S2.: Cytotoxicity classification of the G30AE, G30LE, negative and positive controls obtained by agar diffusion test.

ILLUSTRATION OF THE SAMPLE ON PETRI PLATE

Samples

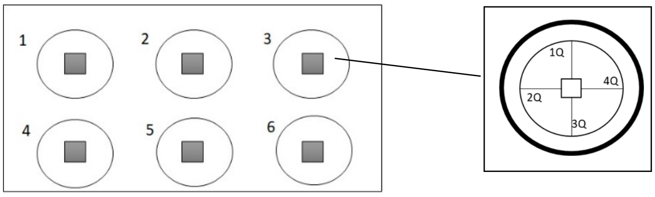

PLATE READING - SAMPLES

	Hole/Q*	1Q (mm)	2Q (mm)	3Q (mm)	4Q (mm)	A**(cm)	OA*** (cm):	Results
**G30AE**	1	0	0	0	0	0	0	☒ Satisfactory<br>☐ Unsatisfatory<br>☐ Repeat
	2	0	0	0	0	0		
	3	0	0	0	0	0		
**G30LE**	4	0	0	0	0	0	0	☒ Satisfactory<br>☐ Unsatisfatory<br>☐ Repeat
	5	0	0	0	0	0		
	6	0	0	0	0	0		

PLATE READING - Controls

	Hole/Q*	1Q (mm)	2Q (mm)	3Q (mm)	4Q (mm)	A**(cm)	OA*** (cm):	Results
**Positive Control**	1	8	8	9	9	0.85	0.875	☐ Satisfactory<br>☒ Unsatisfactory<br>☐ Repeat
	4	10	9	9	8	0.90		
**Negative Control**	2	0	0	0	0	0	0	☒ Satisfactory<br>☐ Unsatisfactory<br>☐ Repeat
	5	0	0	0	0	0		
**Blank**	3	–	–	–	–	–	–	☐ Satisfactory<br>☐ Unsatisfactory<br>☐ Repeat
	6	–	–	–	–	–		

*Quadrant **Average ***Overall Average
