# Supplementary figures and images for: Chitosan-PEG Gels Loaded with Jatropha mollissima (Pohl) Baill. Ethanolic Extract: An Efficient and Effective Biomaterial in Hemorrhage Control
Source: Pharmaceuticals (Basel). 2023 Oct 3;16(10):1399. doi: 10.3390/ph16101399 (PMC10609772; doi:10.3390/ph16101399)

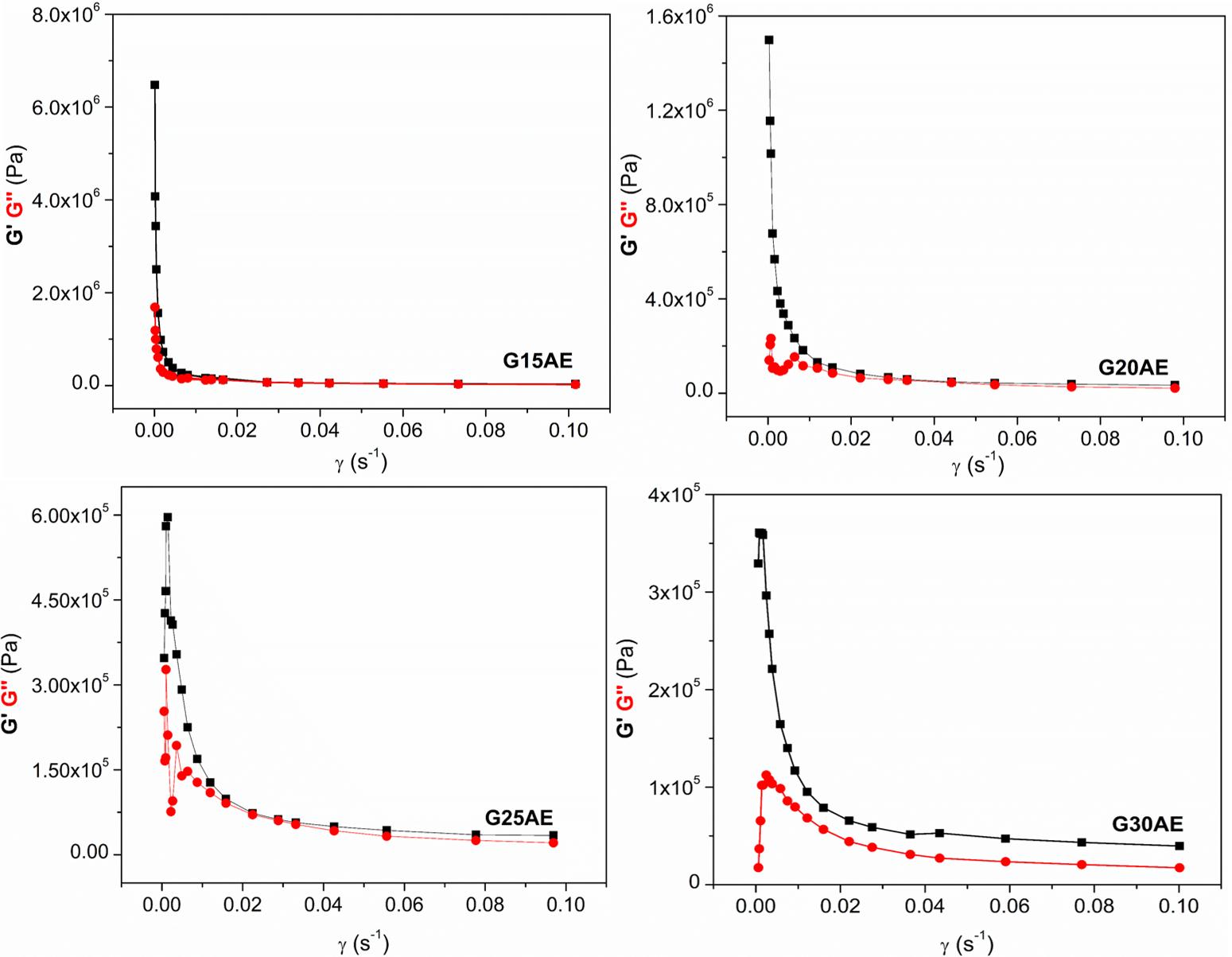

Supplement: Supplementary file 1 [file pharmaceuticals-16-01399-s001.zip › Figure 10S.tif]

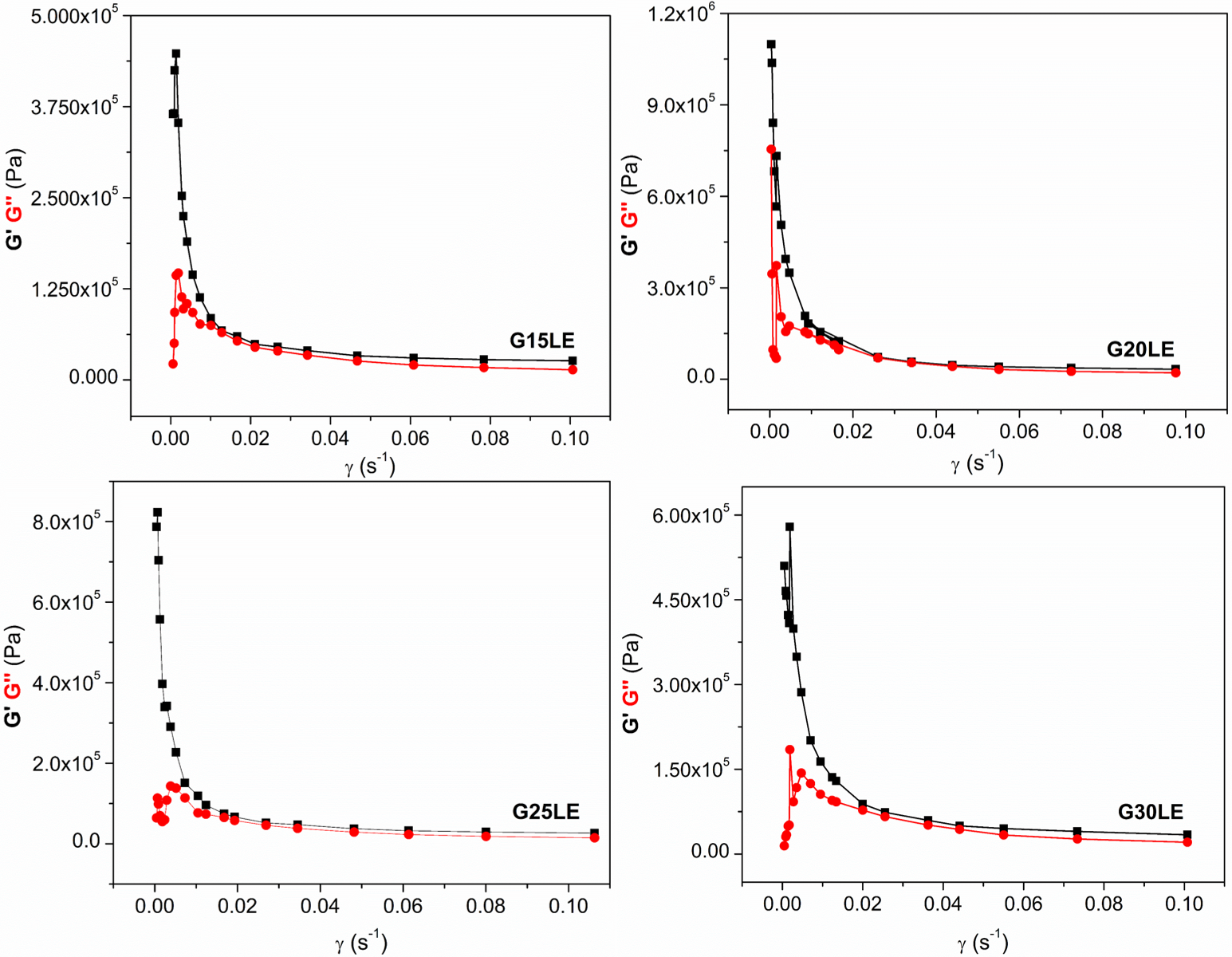

Supplement: Supplementary file 1 [file pharmaceuticals-16-01399-s001.zip › Figure 11S.tif]

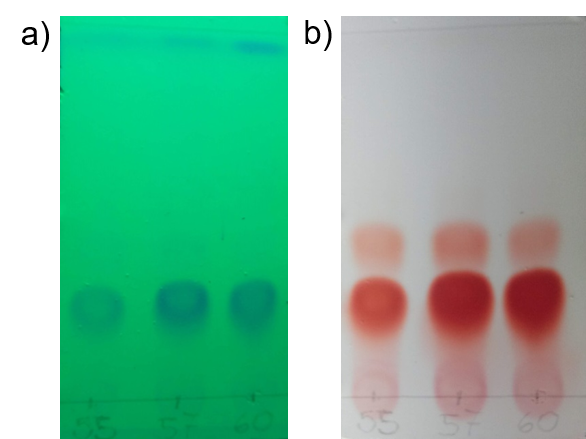

Supplement: Supplementary file 1 [file pharmaceuticals-16-01399-s001.zip › Figure 1S.tif]

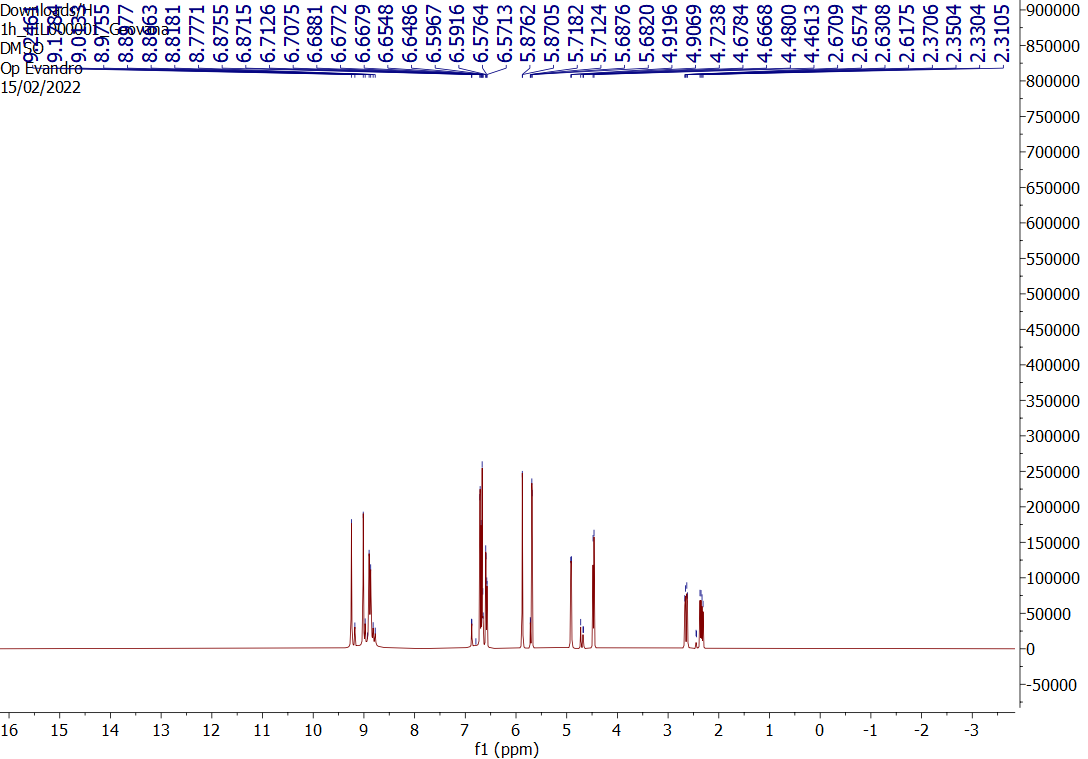

Supplement: Supplementary file 1 [file pharmaceuticals-16-01399-s001.zip › Figure 3S.tiff]

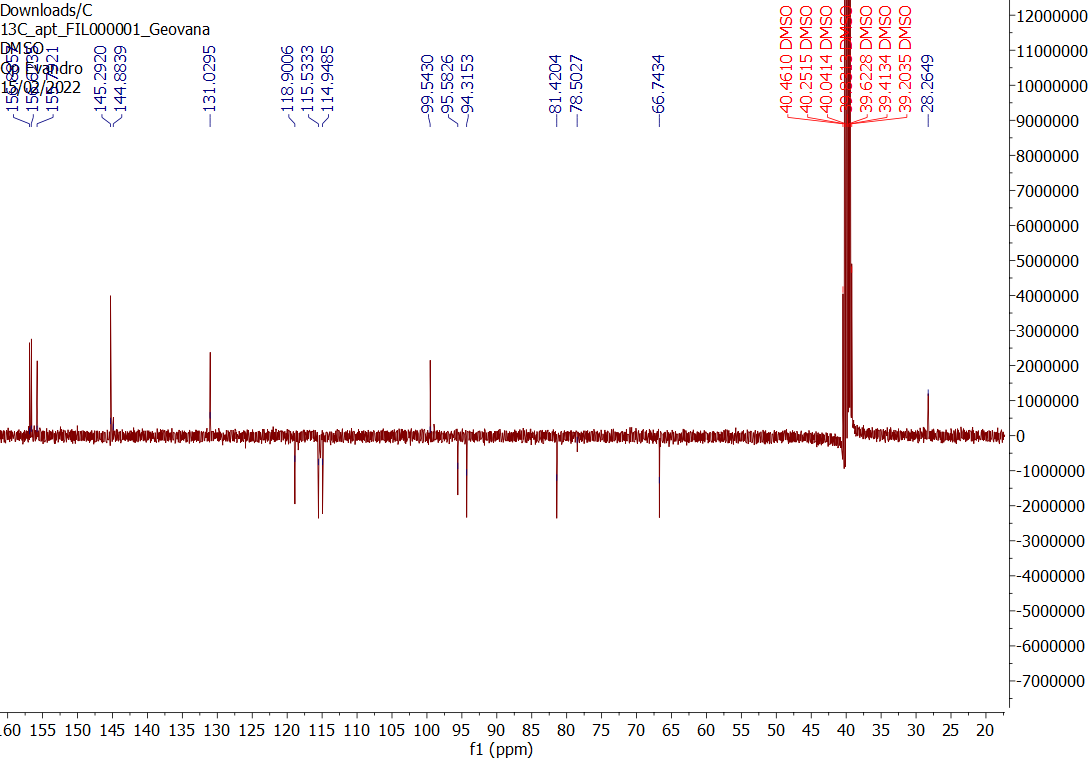

Supplement: Supplementary file 1 [file pharmaceuticals-16-01399-s001.zip › Figure 4S.tiff]

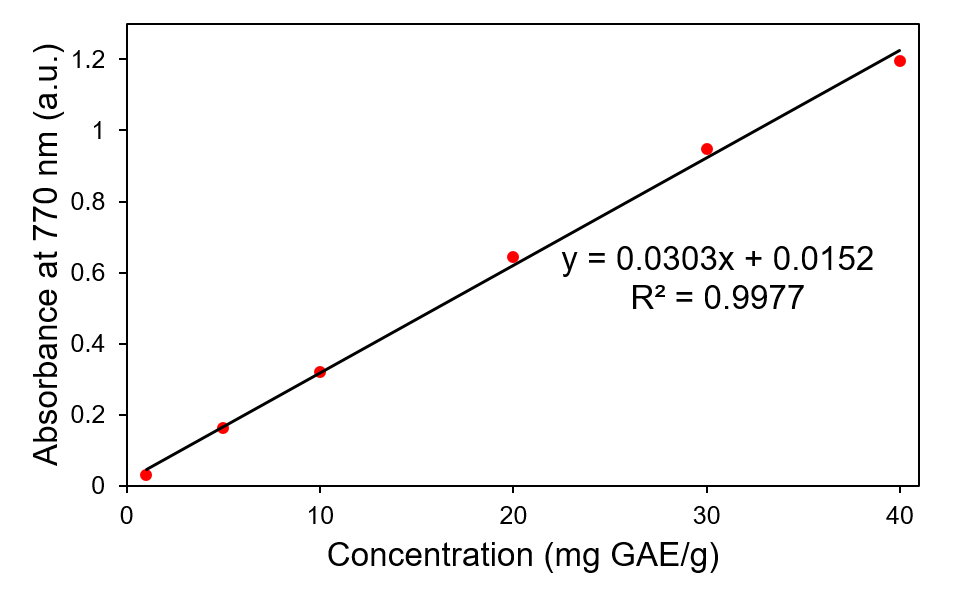

Supplement: Supplementary file 1 [file pharmaceuticals-16-01399-s001.zip › Figure 6S.tif]

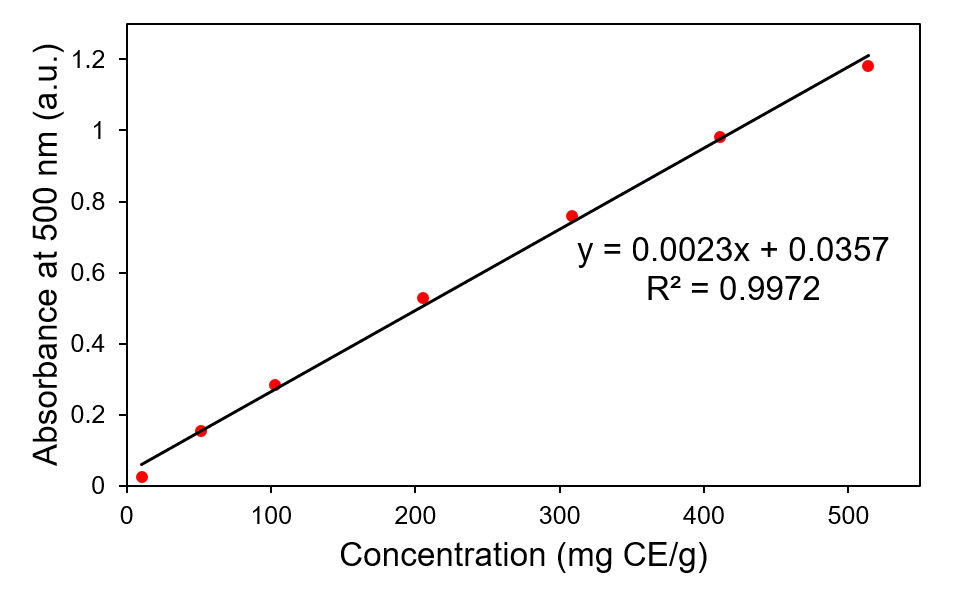

Supplement: Supplementary file 1 [file pharmaceuticals-16-01399-s001.zip › Figure 7S.tif]

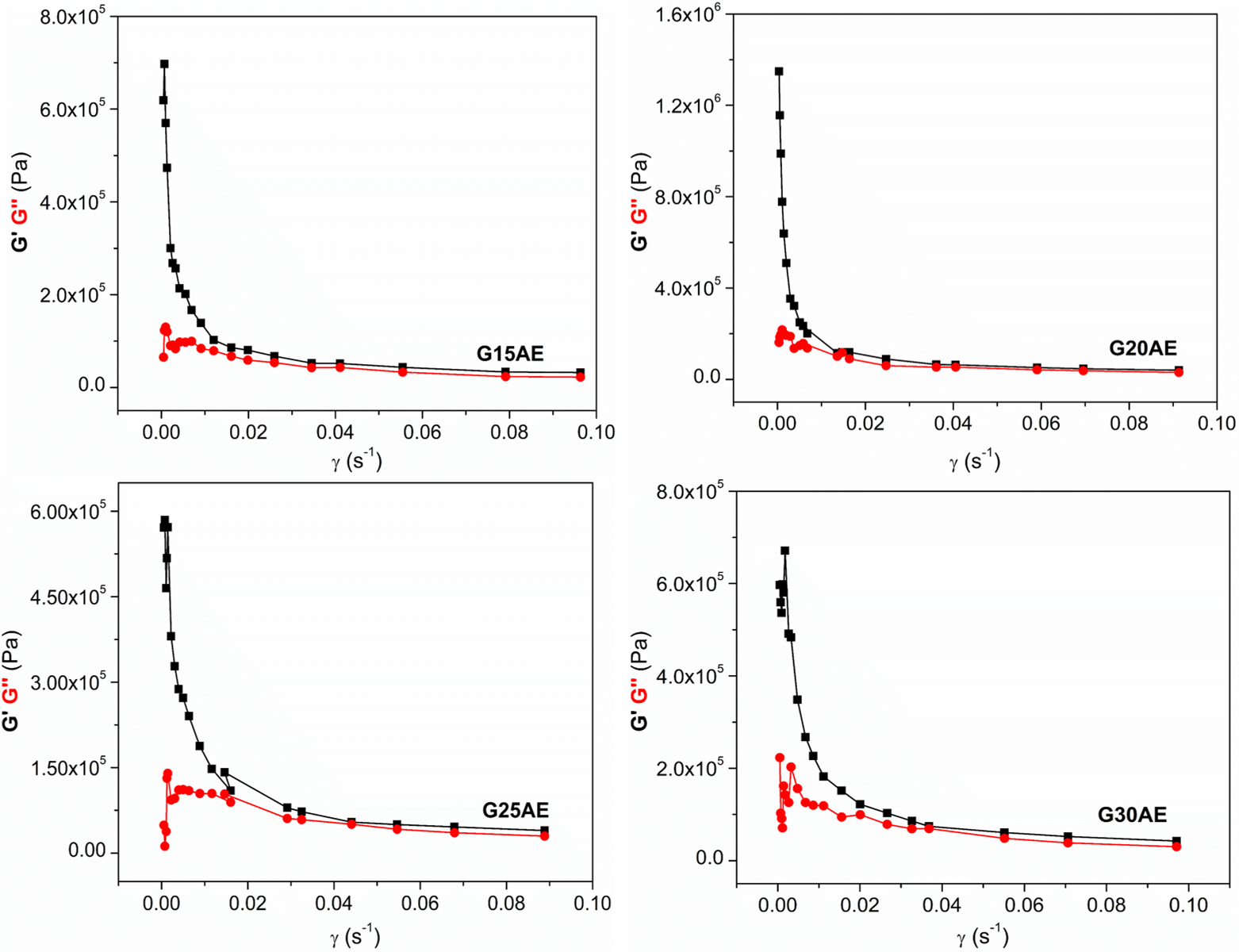

Supplement: Supplementary file 1 [file pharmaceuticals-16-01399-s001.zip › Figure 8S.tif]

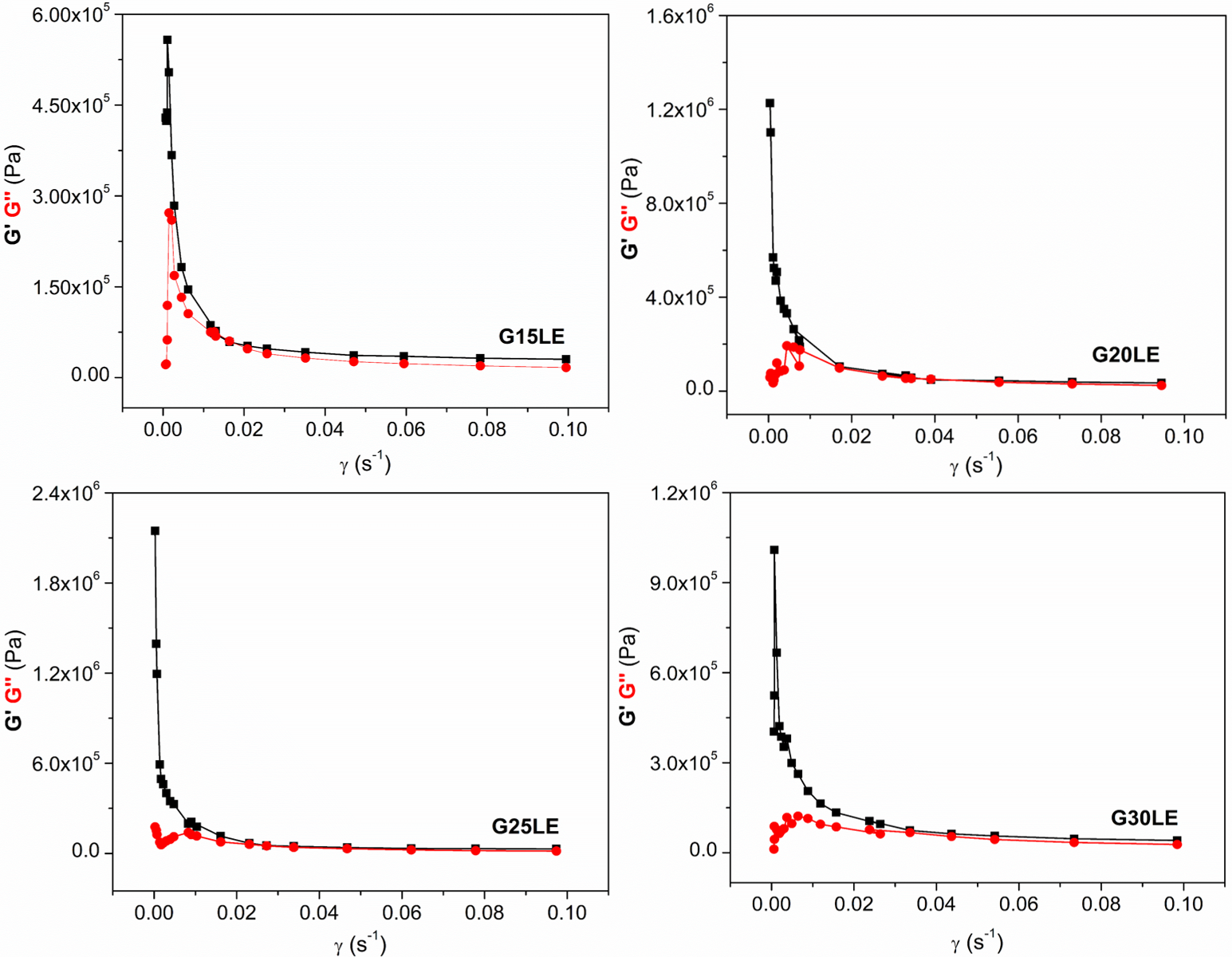

Supplement: Supplementary file 1 [file pharmaceuticals-16-01399-s001.zip › Figure 9S.tif]
